# Investigating the Conformational Diversity of the TMR‑3 Aptamer

**DOI:** 10.1021/jacs.5c04576

**Published:** 2025-05-13

**Authors:** Maximilian Gauger, Elke Duchardt-Ferner, Anna-Lena J. Halbritter, Thilo Hetzke, Snorri Th. Sigurdsson, Jens Wöhnert, Thomas F. Prisner

**Affiliations:** † Institute of Physical and Theoretical Chemistry and Center of Biomolecular Magnetic Resonance, Goethe University Frankfurt, Max-von-Laue Str. 7, Frankfurt am Main 60438, Germany; ‡ Institute for Molecular Biosciences, Germany and Center for Biomolecular Magnetic Resonance (BMRZ), Goethe University Frankfurt, Max-von-Laue-Str. 9, Frankfurt 60438, Germany; § Science Institute, 63541 University of Iceland, Dunhaga 5, Reykjavik 107, Iceland

## Abstract

Aptamers are a class of in vitro selected small RNA motifs that bind a small-molecule ligand with high affinity and specificity. They are promising candidates for the regulation of gene expression in vivo and can aid in further understanding the interaction of RNA with small molecules and conformational changes that may occur upon ligand binding. The TMR-3 aptamer was selected via systematic evolution of ligands by exponential enrichment (SELEX) and binds the fluorophores tetramethylrhodamine (TMR) and 5-carboxy-tetramethylrhodamine (5-TAMRA) with nanomolar affinity. The three-dimensional structure of the TMR-3 aptamer complex with 5-TAMRA was previously determined using liquid-state NMR. By combining the existing NMR restraints with long-range PELDOR distance and orientation information, a broad structural ensemble was generated. From this broad ensemble, a subset of structures was selected by globally fitting orientation-selective PELDOR data from multiple frequency bands. The subensemble represents the conformational variety resulting from the dynamics of the complex. The overall structure of the three-way junction, previously reported by NMR experiments, is retained in the ensemble of the bound state and we were additionally able to characterize the fluctuation of the different stems of the aptamer. Furthermore, in addition to the ligand-bound state we could access the unbound state of the TMR-3 aptamer which was previously uncharacterized. The unbound state of the aptamer is much more structurally diverse, compared to the ligand-bound state. A significant fraction of the ensemble of the unbound state strongly resembles the ligand-bound state, indicating that the ligand-bound state is preformed, which further suggests a conformational-capture ligand-binding mechanism. Apart from the conformations that resemble the ligand-bound state, distinct conformational states which are not present in the presence of the ligand, were successfully identified.

## Graphical abstract

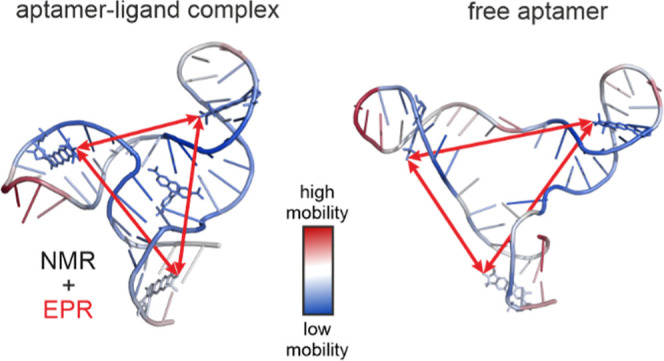

## Introduction

Characterizing the structure, conformations, and dynamics of RNA is paramount to understanding its function. Structure refers to the spatial arrangement of atoms within the RNA, conformations are different spatial arrangements of atoms in a molecule resulting from rotations about single bonds, and dynamics refer to the time-dependent changes between different conformations. To probe RNA on different time scales and with different spatial resolution, a number of techniques have been developed.

Established methods in structural biology such as cryogenic electron-microscopy,^1–4^ X-ray crystallography,^5,6^ and nuclear magnetic resonance (NMR) spectroscopy^7–9^ can provide structural insights with high spatial resolution. The large conformational diversity of RNA and the challenges in sample preparation and crystallization regularly pose challenges to the application of these methods.

In the context of this work, we want to highlight a combination of nuclear magnetic resonance (NMR) spectroscopy with pulsed electron paramagnetic resonance (EPR) spectroscopy for the study of RNA.

NMR spectroscopy can determine RNA structures with high resolution through many short-range interactions in the subnanometer range.^7–9^ Because these interactions are typically averaged due to fast conformational dynamics and tumbling of the RNA molecules, most of the restraints reflect the main average structure of the molecule in solution. In addition to the structural insights, the dynamics of local and, under certain conditions, large-scale domains can be probed by different dedicated NMR techniques.^10–14^

EPR spectroscopy is a powerful technique to observe the structural ensemble of paramagnetically labeled biomolecules.^15–21^ Using pulsed dipolar spectroscopy experiments such as pulsed electron–electron double resonance (PELDOR) spectroscopy,^22^ also called double electron–electron resonance (DEER) spectroscopy,^23^ distances between paramagnetic centers in the range of 1.5 to 16 nm^24^ can be measured. To access shorter distances with EPR spectroscopy, electron nuclear double resonance (ENDOR) spectroscopy can be used to probe the local environment of a paramagnetic center up to 1.5 nm which has found recent success in integrated structural biology.^25–30^ Since pulsed EPR experiments are typically carried out in frozen solutions, an ensemble of structures is captured, which represents the ensemble at the freezing temperature. This provides access to distributions of distances which can resolve ensembles of structures that fluctuate around a preferred conformation or multiple distinct conformational states. PELDOR distance distributions in the 1–10 nm range, reporting on the ensemble of the biomolecules, are ideally suited to complement high-resolution techniques such as X-ray crystallography or NMR spectroscopy that generally report a time-averaged structure.^31–37^ In the work presented here, we highlight the potential of combining long-range PELDOR EPR restraints with short-range NMR restraints. We show that the additional PELDOR distance restraints can extend the characterization of secondary and tertiary structure motifs of NMR structures by their internal flexibility and dynamics.

As there are typically no paramagnetic centers in RNA, paramagnetic spin labels have to be introduced synthetically for EPR investigations.^38–41^ In this project we chose to use the cytidine analogue Çm spin label (Figure 1A).^42^ When introduced into a helical region opposite to guanine, Çm forms a base pair with three hydrogen bonds, analogous to a Watson–Crick C–G base pair. Thus, Çm is rigidly incorporated with negligible internal degrees of freedom,^29^ allowing the measurement of very precise distances between two spin labels, incorporated into the same RNA. Additionally, information about the relative orientation of the spin labels can be obtained from multifrequency/multifield experiments.^43–46^ This is achieved by applying selective pulses at specific frequencies or field positions in the PELDOR experiment, which selectively excite different orientations of the anisotropic g- and hyperfine-tensors of the labels with respect to the external magnetic field. If both spin labels in the PELDOR experiment are rigidly incorporated, their relative tensor orientations are related to the dipolar axis system. Through selective excitation, a non-statistical distribution of θ (the angle between the dipolar vector and the magnetic field) is selected which gives rise to frequency- and field-dependent PELDOR time traces. Since the orientation of Çm is determined by the conformation of the biomolecule which Çm is attached to, these orientation-selective PELDOR experiments can provide more detailed structural information such as the angle between two helices.^33,47,48^ The long-range distance and orientation restraints provide complementary information to, e.g., short-range interactions measured by NMR spectroscopy^32,35–37^ and provide additional information about the conformational variability of the RNA.

**Figure 1:**
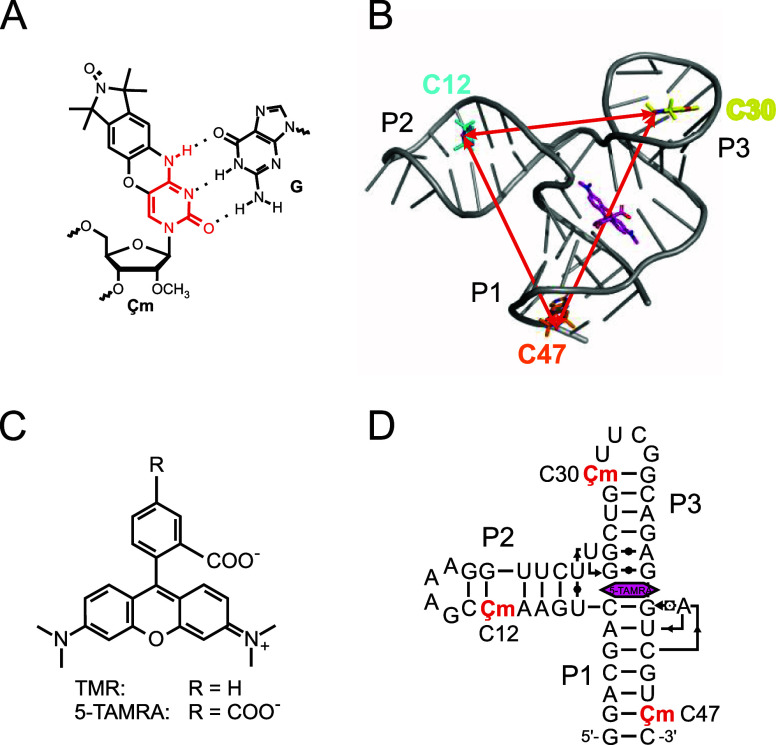
(A) The rigid spin label Çm in the context of a Çm-guanine (G) base pair. Hydrogen bonds are indicated. The part of the structure that resembles the cytosine nucleobase is highlighted in red. (B) Three-dimensional structure of the TMR-3/5-TAMRA complex determined by NMR spectroscopy (pdb: 6gzk).^49^ The RNA is shown in gray cartoon representation, 5-TAMRA in stick representation with the carbon atoms in pink and the nitrogen atoms in blue. Çm spin labels were applied by superimposing the spin label structure onto the respective nucleotide. (C) Structure of TMR and 5-TAMRA. (D) Secondary structure of the TMR-3 aptamer in complex with the ligand 5-TAMRA (pink hexagon) in Leontis-Westhof notation.^49,50^ The positions C12, C30 and C47, where spin labels were introduced are marked with Çm.

There are different approaches for analyzing orientation-selective PELDOR data. If high-resolution modeling data or molecular dynamics (MD) simulations are available, it is possible to calculate orientation-selective PELDOR data from the predicted structures and associated spin label orientations. The simulations can then be compared directly with the experimental data, which can help to validate the MD simulations and at the same time explain the PELDOR data.^29,51–53^ If no prior model with structural and dynamical information exists, simulations of spin label pairs with little or no conformational restraints can be generated. From these spin label pairs, orientation-selective PELDOR data can be calculated and used to fit the experimental data.^54,55^ A drawback of this approach is the inherent ambiguity of these experiments which arise from the symmetry properties of the tensors describing the spin interactions. If some prior model or structural information is available, a broad ensemble of structures with associated spin-label orientations can be modeled. A subset of structures can then be selected by fitting the experimental data.^32^

In previous studies, Çm^42^ (Figure 1A) and its deoxy-analogue Ç^56^ have been used to study the conformational diversity of different RNAs and DNAs. The conformational dynamics of RNA^29,45^ and DNA^32,43,51,57,58^ duplexes have been studied extensively using PELDOR and more specifically orientation-selective PELDOR in combination with molecular modeling and MD simulations. The unrestricted fitting methodology has also been applied to study more complex structures including an RNA^48^ and a DNA^33^ aptamer. Here, we will expand on these preceding studies by studying an RNA aptamer which consists of a three-way junction. We employ an approach in our data analysis which yields tangible structures. This allows us to assess the structural diversity of the aptamer. For the first time, we present conformational ensembles for the ligand-bound state as well as the free state of the TMR-3 aptamer.

RNA aptamers are a class of relatively small RNA motifs which are selected in vitro by Systematic Evolution of Ligands by Exponential Enrichment (SELEX) to bind with high affinity and specificity to their respective small-molecule-ligand.^59,60^ In many cases, the RNA undergoes a conformational change upon binding to the ligand.^61^ Some aptamers are artificial switches which can regulate the expression of proteins in vivo by undergoing a ligand-induced conformational change.^62–66^ This holds great promise for future applications in medicine and therapeutics. Beyond their immediate applications, RNA aptamers are ideal systems for studying the interaction between RNA and small molecules. This could provide further insights that could improve RNA-based drug discovery and biomolecular engineering.

The tetramethylrhodamine binding aptamer 3 (TMR-3 aptamer, Figure 1B), previously selected by Carothers et al. using SELEX, is one of a handful of candidates that bind the fluorophore tetramethylrhodamine (TMR, Figure 1C) with nanomolar affinity.^67^ TMR was chosen as a ligand because of its cell-permeability, potentially allowing fluorescence studies in cells. The TMR-3 aptamer in particular was selected as it promised the most complex and information-rich structure.^67^ The three-dimensional structure of the TMR-3 aptamer in complex with the water-soluble TMR analogue 5-carboxy-tetramethylrhodamine (5-TAMRA, Figure 1C) was reported previously using solution-state NMR spectroscopy (Figure 1B).^49,68^ The structure consists of a three-way junction with three small helical motifs (P1, P2, P3) with P2 and P3 capped by stable tetraloop motifs. In the ligand-bound state, the ligand 5-TAMRA binds in the junction, stacking between helices P1 and P3, forming a continuous helix. P2 protrudes from this helix at an angle of approximately 90°.^49^ The free state of the aptamer has not been characterized fully due to its high flexibility which is challenging to address by NMR spectroscopy.

The NMR structure derived from Nuclear Overhauser Effect (NOE) distance restraints represents the time-averaged structure of the RNA-ligand complex. However, for many biomolecular systems, conformational plasticity and dynamics play a crucial role in the function and mechanisms involved in cellular processes. In the specific case of an RNA aptamer presented here, the conformational ensemble can give insights into the mechanism of molecular recognition and binding. Binding events are key to all biological processes. Several binding mechanisms have been established, classified, depending on the conformational change that accompanies the binding process. Whereas in a lock-and-key scenario neither of the binding partners changes conformation, an induced fit mechanism is accompanied by conformational changes of one or both binding partners. A special case is conformational selection or conformational capture. In this case, binding occurs to a subpopulation within the free conformational ensemble of one binding partner that resembles the bound state in the absence of the ligand.^69^ Apart from being of fundamental interest, knowledge of the conformational ensemble and resulting knowledge of the binding mechanism can provide routes for pharmacological intervention or optimization.

In this study, we combine the benefits of a high-resolution NMR structure with the long-range restraints from PELDOR spectroscopy to obtain a representative structure ensemble for the TMR-3 aptamer. Whereas an X-ray structure can aid in explaining EPR (PELDOR) data and serve as a structural foundation of the biomolecule in question,^30,31,34^ EPR and NMR data can be used jointly to calculate structures which fulfill both of these data sets and yield structures present in the structure ensemble.^32,35–37^ The ensemble will give us representative insights into the flexibility and dynamics of the aptamer. To obtain the structure ensemble of the TMR-3 aptamer, we chose to follow and adapt the protocol established by Grytz et al.^32^ In the first step, a broad ensemble of structures is calculated by CYANA^70^ using the existing NOE NMR restraints in a less restrained way than for the NMR-structure, together with the newly obtained PELDOR distance distribution widths. From this broad ensemble, a small set of structures is selected by iteratively fitting to orientation-selective PELDOR data, measured at two different frequency bands on the same construct. The resulting subset of structures allows us to refine the positions and orientations of the three helices of the TMR-3 aptamer. Moreover, information on the structure ensemble encoded in the PELDOR experiments adds new insights into the TMR-3/5-TAMRA complex to the existing averaged NMR structure. Through the large conformational diversity of the ensemble obtained from the fitting procedure, the three-way junction observed in the NMR structure is conserved. Helices P1 and P3 are stacked on the ligand 5-TAMRA and helix P2 points away from this helical stack, exhibiting the greatest conformational diversity of the complex.

In addition to the ligand-bound state, we also combined newly recorded PELDOR distance restraints with the hydrogen bonding data of the free aptamer from NMR spectroscopy. This gives access to the conformational ensemble of the free aptamer which we compare to our findings on the ligand-bound state. The helical elements of the RNA-ligand complex are conserved even in the absence of the ligand. The junction is highly flexible in absence of the ligand, leading to a large structural variability in the free RNA. In this highly diverse structure ensemble, we were able to identify multiple conformational states of the free aptamer. PELDOR spectroscopy has the unique advantage that distance restraints can be obtained regardless of the flexibility of the system. Data analysis and interpretation in the case of a highly flexible system is undeniably challenging but can still be informative.

## Results and Discussion

The 48-nucleotide TMR-3 aptamer was labeled with Çm at positions C12 in P2, C30 in P3 and C47 in P1 (Figure 1B,D). Two spin labels were present in each sample, resulting in three constructs (C12C30, C12C47 and C30C47). Overall, we recorded and analyzed PELDOR data at X-, Q- and G-band frequencies of all three constructs in the absence and presence of the ligand 5-TAMRA.

At Q-band (33 GHz, 1.2 T), orientation-selective effects can be assumed to be negligible when recording PELDOR data with an offset of −80 MHz between pump and detection frequencies. The spectral overlap of different g- and hyperfine-transitions leads to an excitation of broad orientation ensembles by both pulses which effectively suppresses orientation selection. This allows the use of conventional data analysis methods such as Tikhonov regularization.^71–73^ Figure 2 shows the distance distributions obtained for the three spin-labeled constructs of TMR-3 in the presence and the absence of 5-TAMRA. In the presence of the ligand, all three labeling pairs exhibit a single well-defined distribution with additional small contributions that do not completely disappear in the data processing (Figure 2, top).

**Figure 2:**
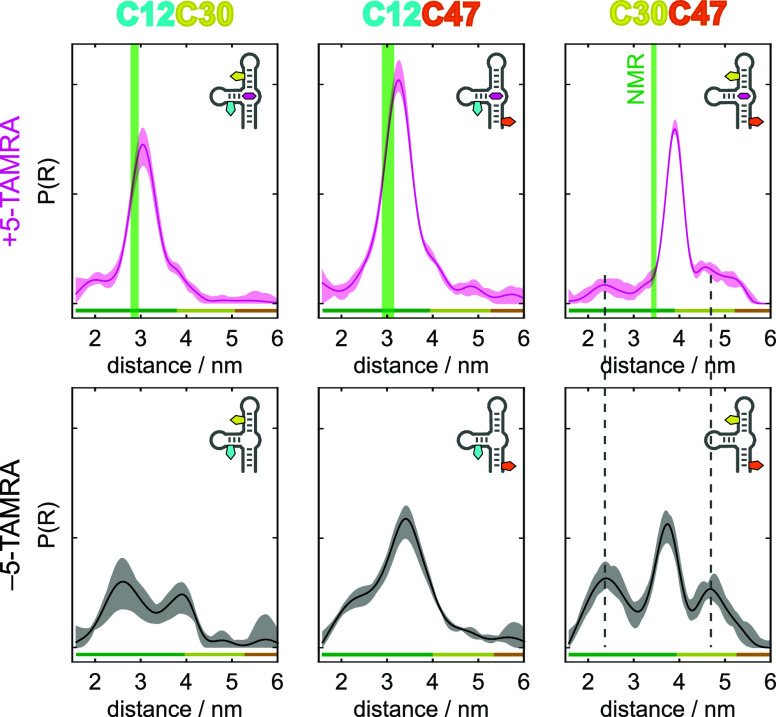
Q-band distance distributions of the three Çm spin labeled constructs of the TMR-3 aptamer in presence (magenta, top) and absence (black, bottom) of the ligand 5-TAMRA. The respective 2σ confidence intervals are indicated by the shaded areas. The bar at the bottom of each distribution indicates the reliability ranges of the distribution (green: reliable shape, yellow: reliable mean and width, orange: reliable mean). *R* = 2.4 nm and *R* = 4.7 nm are marked with a dashed line in the data of C30C47 to illustrate that these distance contributions in the presence of the ligand could stem from residual free aptamer. The distance distributions obtained by applying the structure of Çm to the NMR structure bundle (pdb: 6GZR & 6GZK) are shown in green in the data of the complex.

The most probable distances obtained from the main peaks of the distributions in the presence of 5-TAMRA are 3.0, 3.3, and 3.9 nm for C12C30, C12C47 and C30C47, respectively. These are overall in good agreement with the distances obtained from the NMR structures when the structure of Çm is superimposed on the respective nucleotide (Figure 2, green shaded areas, structures from the pdb: 6GZR & 6GZK). The distances from the NMR structure agree almost perfectly with the most probable EPR distances for C12C30 and C12C47. In contrast, there is a notable discrepancy between the EPR data and the NMR structure for C30C47, although the NMR-predicted C30C47 distance is also still part of the ensemble observed in our EPR experiments. In C30C47, the spin labels are located in helices P3 and P1 that are coaxially stacked onto each other with the ligand 5-TAMRA sandwiched between them. The difference between the C30C47 distance in the NMR-structure bundle (3.4 nm) and the Q-band PELDOR distance (3.9 nm) is 0.5 nm. It is possible that in the NMR-structural analysis, which is based on short-range NOE-distance information, the twist or winding of the helices is not fully characterized. The winding of the helix would affect the spin-label distances, which could explain the observed discrepancy. Another explanation could be a small local reorganization and small internal dynamics of the Çm spin label, as recently described.^29^ Such effects could be amplified at position C47, due to its proximity to the blunt end of the aptamer. This region could have a higher degree of mobility. However, the discrepancy observed here is about four times larger than what was observed when introducing Çm into RNA duplexes.^29^

The full width at half-maximum (fwhm) values of 0.7, 0.7, and 0.5 nm for C12C30, C12C47 and C30C47, respectively, suggest that the aptamer–ligand complex is very well structured with some structural fluctuations around a preferred conformation. The fwhm of the distance distribution of C30C47 in the presence of 5-TAMRA is comparable to the values obtained for a small duplex RNA, labeled with Çm^29^ and suggests that the stack of helices P1 and P3 is less dynamic as compared to helix P2 (C12) which agrees with expectations derived from the NMR structure, where P2 points away from the continuous helix stack P1/P3. Compared to the distance distributions observed in the PELDOR experiments, the structural fluctuations reported in the bundle of the 20 structures from liquid-state NMR are significantly smaller (Figure 2, green shaded areas, structure entries from the pdb: 6GZR & 6GZK). This is not unexpected since the PELDOR experiments were carried out in frozen solution, where the conformational ensemble contains the variety of structures that the aptamer takes on. In contrast, the liquid-state NMR data are averaged over the time scale of the experiment and thus report a mean structure in the presence of a dynamic conformational equilibrium.

Figure 2 (bottom, black) additionally shows the distance distributions obtained for the three labeled constructs of TMR-3 in the absence of the ligand. All three distance distributions are broader than their respective counterparts in the presence of 5-TAMRA, indicating that the free aptamer is more flexible than the aptamer–ligand complex. This finding is well in line with the NMR spectra of the free TMR-3 RNA which showed a significantly lower degree of structuring, especially around the three-way junction of the RNA (see Figure S12).^49^

All three distance distributions of the free aptamer contain additional distance components. The most probable distances in the distributions of C12C47 and C30C47 are almost identical in the bound and unbound state. However, the fwhm of the main distance is larger for the free aptamer signifying a larger structural variability around a common average. The distribution of C12C47 in the absence of the ligand has an additional shoulder at a smaller distance around 2.5 nm. The distribution of the free C30C47 has well-separated distance contributions at 2.4 and 4.7 nm. These coincide precisely with the small additional contributions which are observed in the presence of 5-TAMRA (Figure 2; gray dotted line). This led us to assume that a small fraction of free aptamer is present even in the presence of the ligand (more data of C30C47 under different conditions is shown in the Supporting Information, Figure S3). In contrast to the distances obtained for the unbound C12C47 and C30C47, the most probable distance in the distance distribution of C12C30 does not coincide with the most probable distance of the bound form and shows a bimodal behavior with distance maxima at 2.6 and 3.9 nm. The bimodal distribution of C12C30 in the absence of the ligand suggests that helix P2 has a slight preference for being positioned close to helix P1 (long C12C30 distance) or helix P3 (short C12C30 distance) in the absence of a fully structured binding pocket. This can also be rationalized by the shoulder around 2.5 nm in the distribution of C12C47 in the absence of 5-TAMRA, which would appear when P2 (C12) is close to P1 (C47).

To summarize the changes between the distance distributions of the free aptamer and the aptamer–ligand complex qualitatively, helix P2 (spin label position C12) is more flexible in the absence of the ligand, leading to broader distributions. Also, helices P1 and P3 which form a continuous ligand-mediated helical stem in the complex, seem to adopt either an elongated or a collapsed conformation in the free form, leading to the observed long and short distances, respectively. Since the main distance distribution of the unbound aptamer coincides with the distribution of the RNA-ligand complex, particularly for C30C47, we conclude that the structure of the complex is preformed, even in the absence of the ligand. This suggests a conformational-capture ligand-binding mechanism for the TMR-3 aptamer. Similar observations have been made for the neomycin-binding and fluoride-binding riboswitches.^30,74^ A more in-depth structure interpretation is described below.

For both the aptamer–ligand complex (Figure 3) and the free aptamer (Figure 4), we observe orientation-selection in the PELDOR data recorded at X- (9.4 GHz, 0.3 T) and G-band (180 GHz, 6.4 T). We can observe changes of the oscillation frequency and the weighting between ν_dd_ and 2ν_dd_. These are the frequencies that are observed when the interspin vector *R* is perpendicular (θ = 90°) or parallel (θ = 0/180°) to the magnetic field direction, respectively (ν_dd_ = *D*_dip_·(3 cos^2^ θ – 1)·*r* ^–3^; *D*_dip_ = 52 MHz/nm^3^). Furthermore, the dampening of the oscillations, which is a result of the weight between ν_dd_ and 2ν_dd_, and the modulation depth recorded at different frequency offsets or field positions, depend on the orientation of the spin labels.

**Figure 3:**
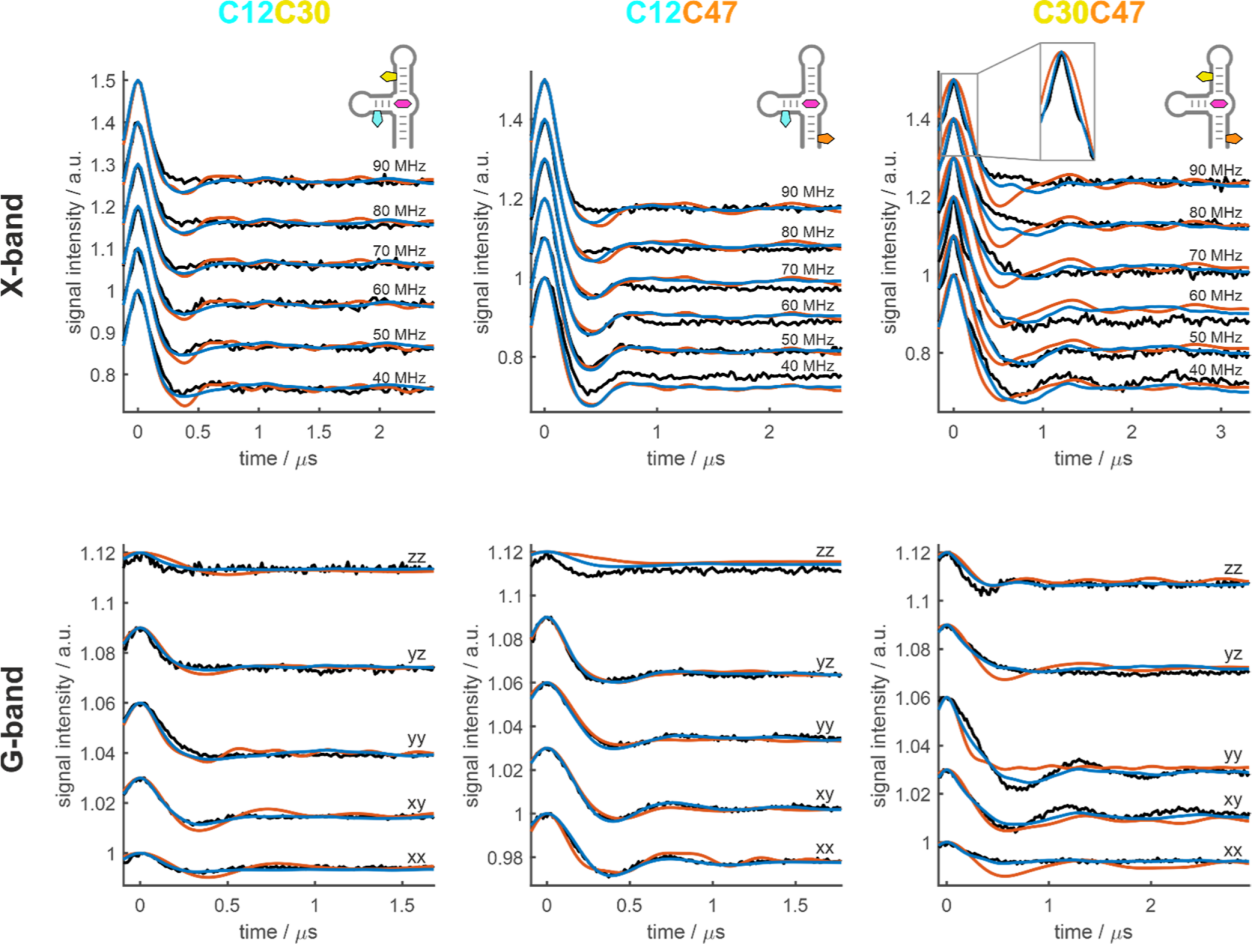
Orientation-selective PELDOR data (black) of the three Çm spin-labeled constructs of the TMR-3 aptamer in the presence of the ligand, measured at X-band (top; 9.4 GHz, 0.3 T) and G-band (bottom; 180 GHz, 6.4 T) compared to the result of the fit using different structure ensembles (blue and orange). Orange: The experimental data were fit by selecting 50 structures from a subensemble of approximately 1000 structures with the lowest target function values. Blue: The experimental data were fit using 60 structures in total. 50 ligand-bound structures were selected by fitting to the experimental data of the aptamer–ligand complex. After the fourth iteration (70% RMSD convergence), 10 structures with a C30C47 distance below 3 nm were taken from the fit ensemble of the free aptamer (see Figure 4) and were added to the fitting procedure of the aptamer–ligand complex. The insert in the X-band data set of C30C47 (top, right) highlights the region close to *t* = 0 μs. All time traces are shown with an offset to improve readability.

**Figure 4:**
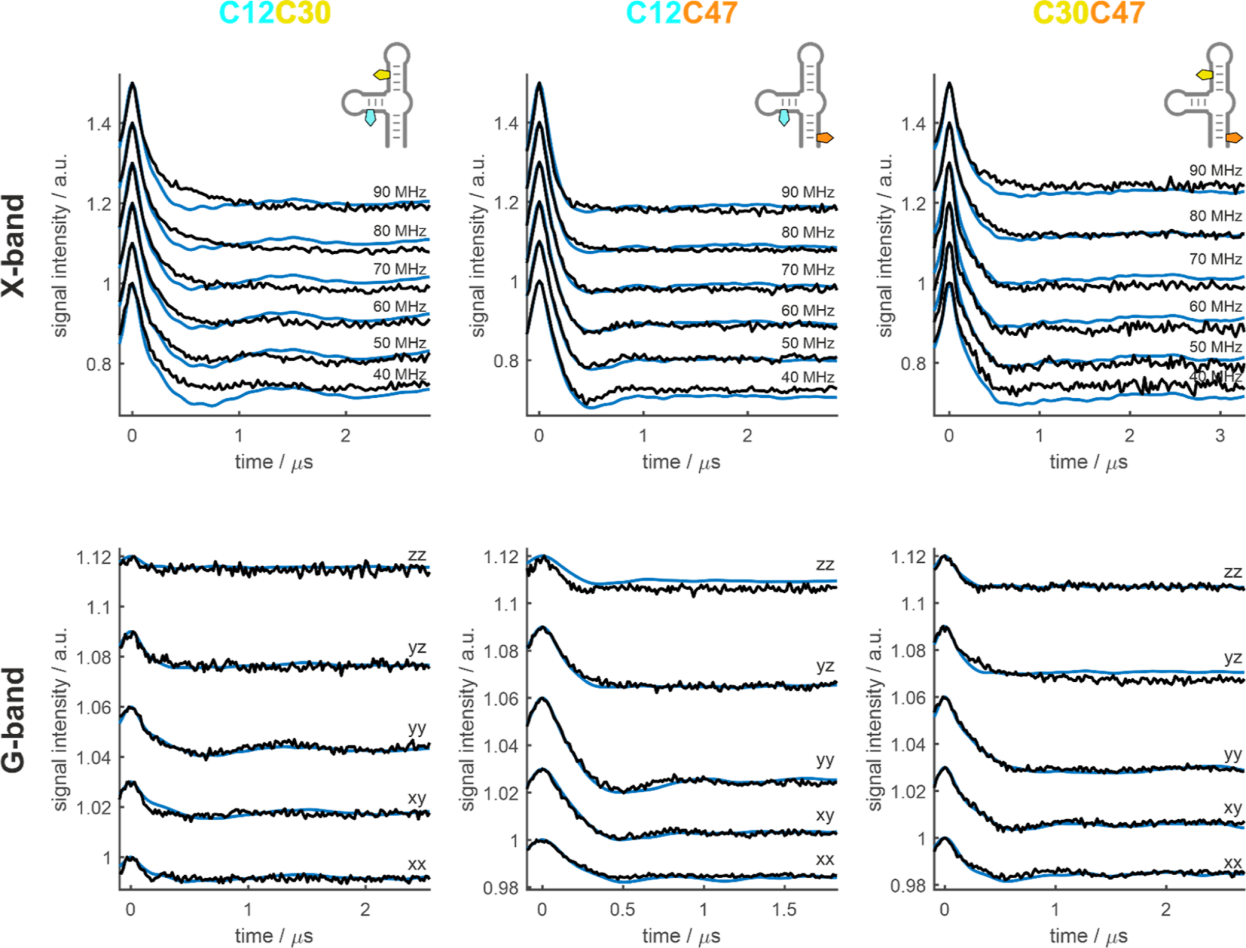
Orientation-selective PELDOR data measured at X-band (top; 9.4 GHz, 0.3 T) and G-band (bottom; 180 GHz, 6.4 T) for the three Çm spin-labeled constructs of the TMR-3 aptamer in absence of the ligand (black). Fit of the experimental data, obtained by selecting 50 structures from a broad ensemble of 7000 structures is shown in comparison (blue). The time traces are shown with an offset to improve readability.

The orientation-selective PELDOR data of the ligand-bound state exhibits well-defined oscillations for all three spin label pairs. From the X-band data of C30C47, the most information can be extracted by visual analysis. The data is characteristic for spin labels where the *z*-axes of the spin labels (normal vector to the molecular plane of Çm) are nearly parallel to the interspin vector. The most pronounced oscillation with the dipolar frequency ν_dd_ can be observed on the trace recorded with 40 MHz offset between the pump and detection frequencies. The oscillation becomes less pronounced with increasing offset and is almost fully dampened at 70 MHz offset. At 90 MHz offset the double frequency 2ν_dd_ is clearly observed. Since the X-band nitroxide spectrum is dominated by the hyperfine *z*-component Azz, the orientation selection can be understood by considering only the orientation of the spin label *z*-axes with respect to the interspin vector. The strongest selection of Azz occurs at the edge of the nitroxide spectrum (the 90 MHz offset trace). When the spin labels are nearly parallel and their *z*-axes are nearly parallel to *R* (see Figure S6), a selection of the spin label *z*-axes would coincide with a selection of θ close to 0° (or 180°) resulting in a pronounced oscillation with the frequency 2ν_dd_. This is in line with expectations from the existing NMR structure, where helices P1 (C47) and P3 (C30) are stacked and the superimposed spin labels are close to parallel. The X-band data for C12C30 and C12C47 show the most pronounced oscillations at an intermediate offset of 60 or 70 MHz, which indicates that the spin labels are not parallel, which is again consistent with the NMR structure. These interpretations are based on the assumption that a main conformation exists and that the structures in the ensemble fluctuate around the main conformation.

The G-band orientation-selective PELDOR is not only influenced by the orientation of the spin label *z*-axes, but the in-plane axis orientations can also be resolved. We observe the most pronounced oscillation and largest modulation depth at the *zz* position in the data of C30C47. This is consistent with the conclusion that the spin labels at positions C30 and C47 are nearly parallel, while the other spin label pairs have a large angle between their *z*-axes. At the *zz* field position, both pulses excite the *z*-axes of the spin labels, leading to a very strong selection of θ = 0/180° when the *z*-axes of the labels are parallel to the interspin vector, as for C30C47. The small modulation depth at the xx position in the data for C30C47 further suggests that the spin label *x*-axes are oriented close to 90°, suggesting that the wind/twist of the helices P1 and P3 is slightly different to the twist reported in the NMR structure. The angle between the *x*-axis of the spin labels at C30 and C47, predicted by the NMR structures, is close to 40°. For the other two labeling pairs it is not straightforward to extract qualitative orientation information.

To obtain a more quantitative representation of the X- and G-band PELDOR data, the data was fit using a structural ensemble. As a reference, we first used the 20 published NMR structures to simulate the orientation-selective PELDOR data. The result can be found in Figure S7. The simulation exhibits a notable discrepancy with the experimental data which is to be expected, considering the much narrower distribution of distances predicted by the NMR structures in comparison to the EPR distribution width (see Figure 2, green).

To improve the fit, we combined the published NOE NMR distances,^49,68^ loosened by ±1.5 Å, with the EPR distance distribution widths, to generate a broad structure ensemble of 10,000 structures with CYANA.^70^ As a reference for the achievable fit quality and to stay close to the initial NMR structure, we chose to use only approximately 1000 structures with target function (TF) values that only increase by 10% compared to the TF of the best structure (Figure S10 for TF cutoff). This subensemble already exhibits much more structural variety than the published NMR bundle but still conserves the characteristics of the NMR structure (distance distributions in Figure S8). Using this structure ensemble, the fit for C12C30 and C12C47 is acceptable, especially considering the relatively low conformational variability in the ensemble (Figure 3, orange). However, some major discrepancies between fit and experiment are observed for C30C47. This is not unexpected, because the subensemble is missing both short and long distances (2.4 nm and >4.1 nm for C30C47). The short distance at 2.4 nm in the EPR distance distribution was excluded from the large conformer ensemble of the RNA-ligand complex, because it was attributed to a small percentage of free aptamer in the sample. However, it seems that to improve the fit of the X-band data of C30C47 it will be necessary to reproduce the very steep decline close to *t* = 0 μs (insert in Figure 3, top, right). Also, the maximum distance for C30C47 observed within the subensemble was ∼4.1 nm, which is not sufficiently long and notably shorter than the upper bound that was used and determined by the width of the main contribution in the PELDOR distance distribution (see Figure S8). This shows that the first thousand lowest-TF structures do not contain conformations to adequately describe the ensemble of structures in our EPR samples.

In the next step, we employed the complete ensemble of structures to fit the experimental orientation-selective PELDOR data of the aptamer–ligand complex (Figure S9). The quality of the fit was significantly better than with the small subensemble, yet still inadequate, which we attribute to the missing short distance of C30C47. Further explanation and the fit can be found in the Supporting Information (Figure S9, comparison of the RMSD in Figure S11).

In order to improve the fit of the data of the aptamer–ligand complex, we decided to fit the orientation-selective PELDOR data of the free aptamer and use a small set of the resulting structures with short C30C47 distances as a prior for the fit of the bound state. Figure 4 shows the orientation-selective PELDOR data that was obtained for the free aptamer and the respective fit. Due to the significantly broader distribution of distances in the absence of the ligand, the oscillations and orientation-selective effects are much less pronounced than in the presence of the ligand (compare with Figure 3). However, we still observe changes of the weight between the ν_dd_ and 2ν_dd_ frequencies and changes of the modulation depth at different frequency offsets or field positions. Notably, the data set of C12C47 still exhibits well-defined orientation selection which is not dissimilar to the ligand-bound state. The data of C12C30, specifically the G-band data, shows typical behavior of a bimodal distance distribution with a high- and low-frequency contribution in the time traces.

The structure ensemble of the free aptamer was produced by removing all intermolecular ligand-aptamer NOE restraints as well as base pair hydrogen bonds and NOE distance restraints of residues in base pairs without observable imino proton resonances in the ^1^H NMR spectrum of the free RNA. The latter applied to a number of base pairs adjacent to the three-way junction (Figure S12). The remaining NOE restraints were again loosened by ±1.5 Å and the full width of the PELDOR distributions of the free aptamer were added in the same procedure as for the aptamer–ligand complex (see Table 1 for lower and upper boundaries). The fitting procedure selected 50 conformers from the ensemble of 7000 structures by fitting to the orientation-selective PELDOR data. The fit using these 50 conformers is satisfactory (see Figure 4, blue). The oscillations, the dampened oscillations and the modulation depths are reproduced with a high degree of fidelity for C12C47 and C30C47. There are some discrepancies for C12C30, particularly at X-band. This could mean that the large conformer ensemble cannot fully represent the conformers which are present in the experiment. A further explanation could be that the RMSD-fitting algorithm we use has difficulties fitting the bimodal distribution when the two distance modes are close to each other. Overall, the quality of the fit is satisfactory considering factors such as the noise of the experimental data and the fact that all chosen conformers have to simultaneously agree with three samples measured at two frequency bands.

**Table 1:** Lower and Upper Distance Restraints, Extracted from the Respective PELDOR Distance Distribution^a^

	lower bound (nm)	upper bound (nm)
C12C30 + 5-TAMRA	2.35	3.7
C12C47 + 5-TAMRA	2.5	3.8
C30C47 + 5-TAMRA	3.1	4.65
C12C30	1.5	4.7
C12C47	1.5	5.0
C30C47	1.5	5.3

a These restraints were used in the structure calculations in CYANA.

We then returned to the fit of the ligand-bound state and included 10 conformers with a C30C47-distance below 3 nm that were randomly chosen from 20 conformers from the fit of the free aptamer, all of which fulfill this condition. These were added to the fit ensemble after 4 iteration steps (70% RMSD convergence). 50 conformers of the bound state were chosen by the fitting procedure. The ratio of 10 to 50 conformers approximates the population of the free and bound state in the sample C30C47 in the presence of 5-TAMRA. With a total number of 60 conformers (10 from the fit of the free-aptamer and 50 from the fit of the bound state) the fit is in excellent agreement with the experimental data (Figure 3, blue). The fit of the G-band data is excellent throughout the three data sets and only minor discrepancies of the oscillation shapes (weight between ν_dd_ and 2ν_dd_) in the X-band data are present. In light of the restraints imposed on the fit, namely the necessity to fit the three label pairs simultaneously, we are confident that this represents the optimal fit achievable from a structure bundle, and that the discrepancies are negligible.

In the analysis of the structures that were selected by fitting to the orientation-selective PELDOR data we will first consider the structure ensemble of the aptamer–ligand complex. The structures show the same overall features as the NMR structure. The three-way junction is present in all structures and the stack of helices P1 and P3 with the ligand 5-TAMRA sandwiched between them is intact. Nevertheless, even the bundle resulting from the fit of the ligand-bound aptamer exhibits a considerable degree of structural diversity (see Figure S13). All three helices are highly dynamic, changing their winding and orientation relative to one another significantly throughout the ensemble. Thus, by combining the high-resolution NMR structure with PELDOR distance restraints, we are able to assess the conformational space accessible to the TMR-3 aptamer–ligand complex. To visualize the variability of the structure bundle we aligned the 50 structures of the aptamer–ligand complex by minimizing the RMSD. Figure 5 (top) shows a representative structure from the bundle which was color-coded by the root-mean-square fluctuations (RMSF) between the structures. Since the PELDOR distances report on long-range structural features, our focus lies on the overall structure and its dynamics, rather than the structural variety of individual base pairs. One can clearly observe the largest structural fluctuations at the blunt end and the loops at the end of the individual helices. It is also apparent that helix P2 shows more fluctuations than the other two helices. This is in agreement with the general structural features of the NMR structure of the TMR-3/5-TAMRA complex, where helices P1 and P3 are stacked and are assumed to be more rigid than P2. Interestingly, the region between residues 18 and 24 (highlighted with a red bracket in Figure 5) is significantly more flexible than the rest of the helical regions while residues 6 to 13 are more rigid. This could suggest a twisting motion of the helix P2 around the backbone of nucleotides 6–13.

**Figure 5:**
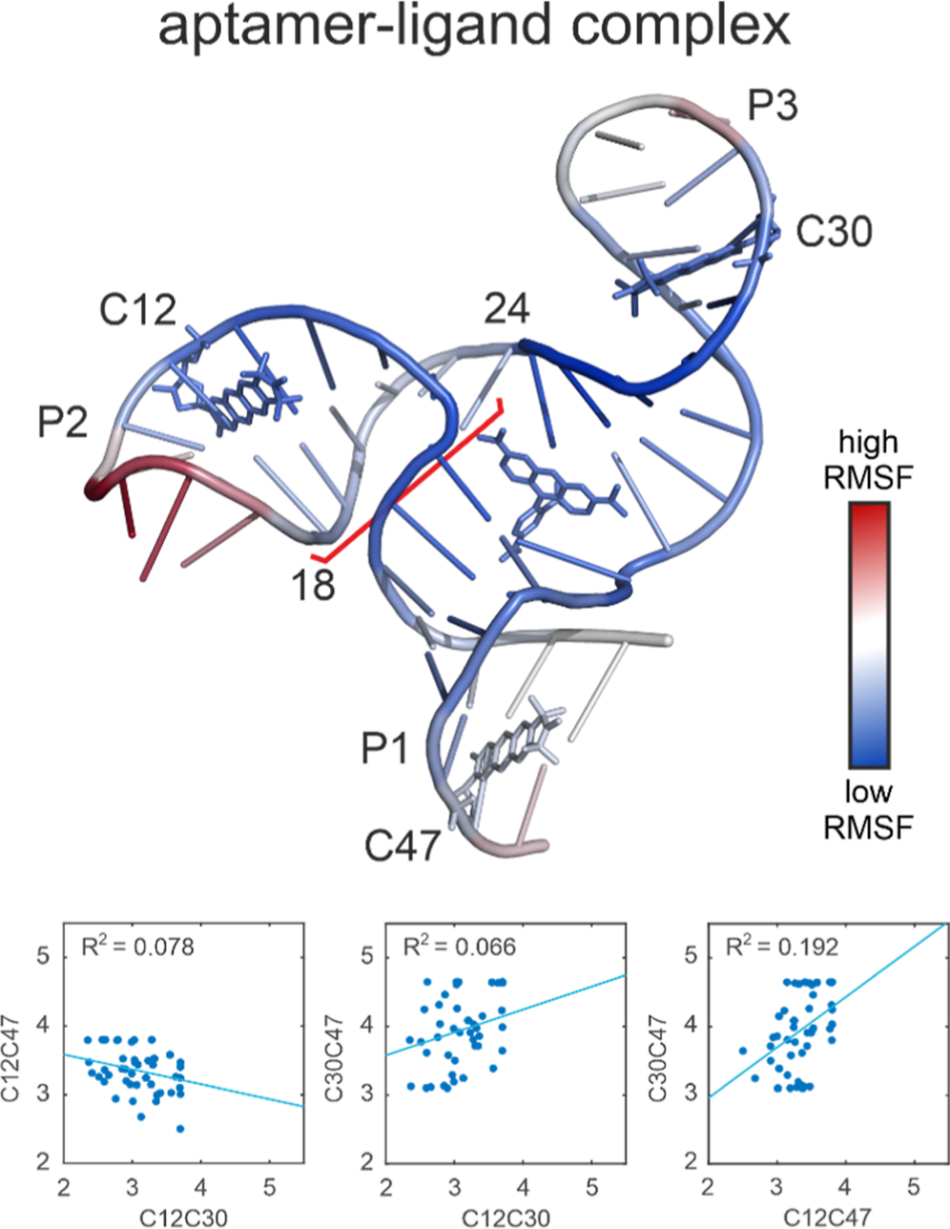
Top: representative structure of the ensemble of 50 structures of the aptamer–ligand complex, obtained by fitting the experimental data. The structure is shown with the RNA in cartoon and the ligand and Çm spin labels in stick representation. The RNA is color-coded with the root-mean-square fluctuation (RMSF) of the respective residue. The region of nucleotide 18 to 24 in helix P2 is highlighted with a red bracket. Bottom: distance correlations of the three label pairs. The distances were extracted from the ensemble of 50 structures, determined by fitting the experimental data. Correlation coefficients are given.

When extracting the interspin distances from the fit ensemble we can observe correlations between the different label-pair distances. First, a negative correlation is observed between C12C30 and C12C47 which shows the motion of helix P2 (Figure 5, bottom). In addition, a positive correlation of C12C47 with C30C47 can be observed. This suggests a breathing motion of the complex where all helices slightly elongate.

The structures in the bundle without the ligand are very diverse (Figure 6). We decided to separate the bundle of structures with a C30C47 distance below and above 3 nm. Around 2/3 of the structures have a C30C47 distance above 3 nm. The structures in this part of the bundle display a high degree of similarity to those observed in the bundle of the bound state. Helices P1 and P3 are also stacked and P2 sticks out to the side. Two conformational states were identified through visual inspection, and their relative populations were quantified (Figure 6). In one state, a hole is present in the ligand binding pocket, yet the structure is intact otherwise. In the second conformation, the binding pocket is collapsed and lacks a defined structure.

**Figure 6:**
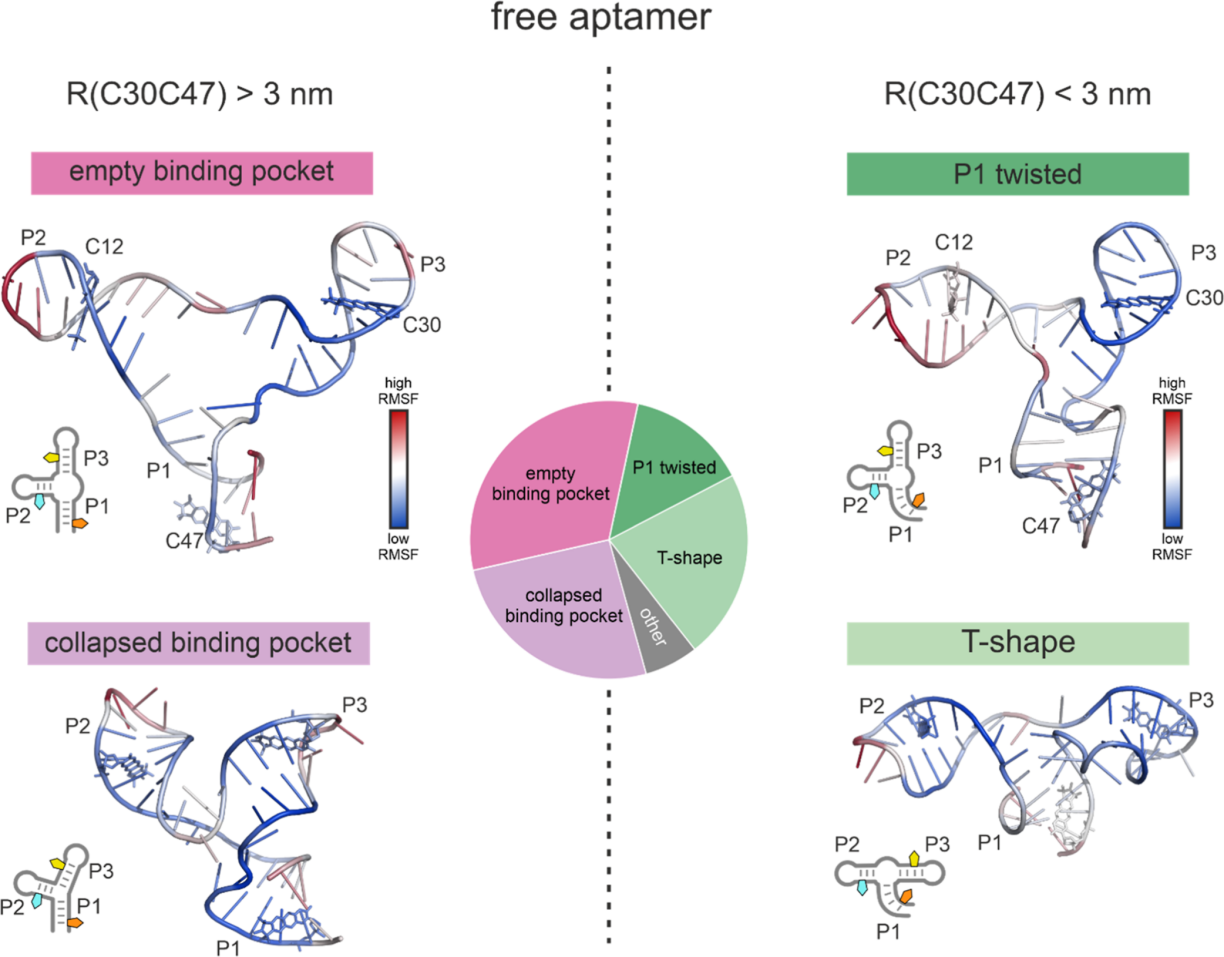
Representative structures found in the structural ensemble obtained from the fit of the orientation-selective PELDOR data of the free (unbound) aptamer with C30C47 distances (R­(C30C47)) above 3 nm (left) or below 3 nm (right). The structures are color-coded with the root-mean-square fluctuation (RMSF) of the respective nucleotide. A simplified schematic of the structures with R­(C30C47) below 3 nm can be found for each structure on the right. The pie chart in the center of the figure depicts the relative population of each state in the fit ensemble.

The structures in the bundle of the free aptamer with a C30C47 distance below 3 nm (population 1/3) can also be roughly classified into two states (Figure 6). The first state looks similar to the bound state with helices P1 and P3 still stacked and an empty binding pocket. In contrast to the state with a 30C47 distance above 3 nm, helix P1 is more strongly twisted such that the spin labels in positions C30 and C47 point toward each other, which leads to a small distance (Figure 6, P1 twisted). This state has a population of around 1/3 of the subset and accounts for the conformers with distances between 2.5 and 3 nm. The second conformation has a population of approximately 2/3 in this subset and the C30C47 distance is between 2.0 and 2.5 nm. In this conformation, helices P2 and P3 are stacked and P1 is oriented orthogonal to the helix stack, forming a T-shape with a long horizontal bar (Figure 6). This conformation is also part of the distance mode around 3.9 nm in the distribution of C12C30.

## Conclusion

In this study we combined precise EPR PELDOR distance and orientation measurements with previously published NMR NOE data^49,68^ to assess the structural diversity of the TMR-3 aptamer. PELDOR measurements were performed on three constructs of the TMR-3 aptamer with spin-label pairs, placed at different positions in the three helical regions of the aptamer. We employed the rigid Çm spin label and performed PELDOR experiments at three frequency bands. The rigid Çm spin label allowed us to obtain well-resolved distance distributions which we used to generate a broad ensemble of structures in conjunction with the existing NOE restraints, loosely following the protocol established by Grytz et al.^32^ From this broad ensemble, a smaller ensemble was selected by globally fitting multifrequency/multifield orientation-selective PELDOR data of the three spin-labeled constructs. Rigid labels such as Çm enable orientation-selective experiments which, in addition to the PELDOR distance restraints, contain information about the mutual orientation of the spin labels attached to the aptamer.

With our approach we were able to obtain a very good fit of the experimental data of the aptamer–ligand complex and a good fit of the data of the free aptamer. By adding a small set of structures of the unbound state to the fit of the aptamer–ligand complex, it was also possible to overcome the challenge of having a small fraction of unbound aptamer in the samples of the complex.

The ensemble of the aptamer–ligand complex, as determined through the fitting procedure, confirms that the overall structural features of the previously reported NMR structure are conserved in a dynamic ensemble. The three-way junction is intact and helices P1 and P3 are stacked, sandwiching the ligand 5-TAMRA. Even with these well-conserved features, a significant degree of flexibility can be observed throughout the structures in the fit ensemble of the complex. The helix stack P1–P3, while intact, is not completely rigid but shows a significant variability of the angle between the helices. The most flexible region, excluding the loops and the blunt end, is between bases 18 to 24 which are located in helix P2. This confirms expectations derived from the static structure where P2 does not participate in the helix stacking.

Our data also provides novel insights into the unbound state of the aptamer. As expected, the free RNA shows significantly more structural variety than the bound state. Specifically through the data obtained from the labeling scheme C30C47 (spin labels in helix P3 and P1 respectively), we separated four distinct states. The majority of the ensemble takes on conformations which are very similar to the ligand-bound state which suggests a conformational-capture ligand-binding mechanism. A smaller fraction of the ensemble takes on different conformations which we were able to broadly classify into two states. The first is again not dissimilar to the ligand-bound state but the binding pocket is collapsed and helix P1 is more curled which yields short C30C47 distances. The second conformation can be described as T-shaped where helices P2 and P3 are stacked and P1 is curled leading again to short C30C47 distances and long C12C30 distances.

By integrating the existing NMR data with our EPR experiments we were able to expand on the high-resolution structure of the TMR-3/5-TAMRA complex by adding long-range distance information of the structural ensemble. It was also possible to observe the ensemble of the free aptamer, which was previously inaccessible to NMR spectroscopy, due to its large flexibility. The combination of NMR and EPR thus enhanced our understanding of the structural variety of the TMR-3 aptamer in its free and ligand-bound form and shows the power of combining these two techniques. It also serves as an example for the great potential of combining multiple experimental methods and structure calculations for the elucidation of the structure and dynamics of biomolecules.

## Methods

### Structure Calculation with CYANA and Fitting Procedure

To generate an ensemble of structures we combined the NMR restraints (NOE derived distances, hydrogen bonding and dihedral angle restraints) that were previously reported with the full width of the main distance peak in the PELDOR distance distribution. The cytidine residues at positions 12, 30, and 47 were replaced by the structure of the Çm label in the aptamer structure to allow direct extraction of the spin label distances and orientation. With this approach, steric clashes of Çm with the RNA structure were avoided during the structure calculation. Three hydrogen bonds were incorporated between Çm-moieties and the respective guanine base-pairing partner of the original C-residue.

A pseudoatom was placed in the center of the N–O bond to represent the position of the electron. The lower and upper distance limits of the main PELDOR distance peaks of the spin-label pairs were assigned to said pseudoatoms (Table 1). The weight of the EPR restraints was adjusted to 10 to account for the strong imbalance in the number of NMR- and EPR-derived restraints. To ensure sufficient conformational variety in the structure bundle, the NOE distances were loosened by ±1.5 Å. 10,000 structures were calculated using CYANA^70^ with target function (TF) values ranging from 0.98 to 144.70 Å^2^ in case of the aptamer–ligand complex. The change of the TF throughout the ensemble, along with the cut-offs applied to create the subensembles used throughout this work can be found in Figure S10.

To generate the ensemble of the free aptamer, all intermolecular ligand-aptamer NOE restraints were removed. Also, the base-pair hydrogen bonds and NOE distance restraints of residues without imino proton resonances (see Figure S12) were removed. This applied in particular to a number of base pairs which are adjacent to the three-way junction. The remaining NOE restraints were again loosened by ±1.5 Å and the full widths of the PELDOR distributions of the free aptamer were added in the same procedure as before. 10,000 structures were calculated using CYANA^70^ with TF values ranging from 2.06 to 260.27 Å^2^.

For every structure and associated spin label orientations it is possible to calculate the pattern of orientation-selective PELDOR time traces. The best structures were selected from the broad ensemble by iteratively fitting to the experimental data. The data of all three spin-label pairs at both frequency bands were fit simultaneously. The weight of the RMSD from fitting the G-band data was scaled up for each label pair by the ratio between the sums of the modulation depth of all recorded time traces for the respective label pair at X- and G-band. This gave the G-band data, which has an order of magnitude lower modulation depth, equal weight to the X-band data and ensures that the data is fit and represented adequately. In general, the modulation depth of the fit was adjusted by a factor for each set of traces. This was done because it is challenging to predict the absolute value of the modulation depth accurately and would require very precise knowledge of, e.g., the resonator profile and the homogeneous line broadening. By not scaling every trace individually, the differences in modulation depth between offsets is kept as a variable, which needs to be predicted correctly by the spin label orientations.

Different subensembles were used throughout this publication. For clarity they are listed and briefly explained here in order of occurrence in the publication:

Minimal bound state ensemble: 50 structures were selected from 1000 structures of the TMR-3/5-TAMRA complex with the lowest TF values (cutoff 1.08 Å^2^). The resulting fit is shown in Figure 3 (orange).

Large bound state ensemble: 50 structures were selected from 8500 structures of the TMR-3/5-TAMRA complex (cutoff 40 Å^2^). The resulting fit is shown in Figure S9 (purple).

Unbound state ensemble: 50 structures were selected from an ensemble of 7000 structures of the unbound TMR-3 aptamer. The resulting fit is shown in Figure 4 (blue).

Refined fit of the bound state: 50 structures were selected from 8500 structures of the TMR-3/5-TAMRA complex (cutoff 40 Å^2^). Ten structures from the fit ensemble of the free aptamer with R­(C30C47) below 3 nm were added to the ensemble after the fourth iteration of the fitting procedure. The resulting fit is shown in Figure 3 (blue).

## Supplementary Material



## Notes

Sample synthesis and preparation, PELDOR experimental setup and procedures, primary PELDOR data and additional simulated and fitted data mentioned throughout the text (PDF)
