# Supplementary material for: Investigating the Conformational Diversity of the TMR‑3 Aptamer
Source: J Am Chem Soc. 2025 May 13;147(20):17497–509. doi: 10.1021/jacs.5c04576 (PMC12100716; doi:10.1021/jacs.5c04576)
Supplement: Supplementary file 1 [file ja5c04576_si_001.pdf]

Supporting Information

Investigating the conformational diversity of the TMR-3 aptamer

Maximilian Gauger,[1] Elke Duchardt-Ferner,[2] Anna-Lena J. Halbritter,[3] Thilo Hetzke, [1] Snorri, Th. Sigurdsson, [3] Jens Wöhnert,[2] Thomas F. Prisner[1]*

[1]Institute of Physical and Theoretical Chemistry and Center of Biomolecular Magnetic Resonance, Goethe University Frankfurt, Max-von-Laue Str. 7, 60438 Frankfurt am Main, Germany

[2]Institute for Molecular Biosciences, Goethe University Frankfurt., Max-von-Laue-Str. 9, 60438 Frankfurt, Germany and Center for Biomolecular Magnetic Resonance (BMRZ), Goethe University Frankfurt, Max-von-Laue-Str. 9, 60438 Frankfurt, Germany

[3]Science Institute, University of Iceland, Dunhaga 5, 107 Reykjavik, Iceland

Contents

1 Sample preparation and description 1
1.1 Oligonucleotide synthesis 1
1.2 Oligonucleotide purification 1
1.3 Analysis of spin-labeled oligonucleotides by MS and EPR spectroscopy 1
1.4 EPR sample preparation 2
2 Experimental details of EPR experiments 2
2.1 X-band PELDOR 2
2.2 Q-band PELDOR 3
2.3 G-band PELDOR 3
3 Additional experimental data 4
3.1 Primary and background-corrected Q-band PELDOR data 4
3.2 C30C47+5-TAMRA – different sample compositions 5
3.3 Background-corrected experimental orientation-selective PELDOR data 6
4 Additional simulated & fitting data 8

1 Sample preparation and description

1.1 Oligonucleotide synthesis

All commercial phosphoramidites, CPG columns, and solutions for oligonucleotide syntheses were purchased from ChemGenes Corp., USA. The solid-phase oligonucleotide synthesis was performed on an automated ASM800 DNA/RNA synthesizer (BIOSSET Ltd., Russia) using phosphoramidite chemistry. The RNA oligonucleotides were synthesized using a trityl-off protocol and with phosphoramidites with standard protecting groups, on a 1 μmol scale (1000 Å CPG columns). Oxidation was performed with *tert*-butylhydroperoxide in toluene (1.0 M). Capping and detritylation were performed under standard conditions for DNA/RNA oligonucleotide synthesis. Unmodified 2′*O*-TBDMS phosphoramidites were dissolved in CH_3CN (0.1 M). The phosphoramidite of **Çm-Bz**[1] (**1**) was dissolved in 1,2-dichloroethane (0.1 M). 5-Benzylthiotetrazole (0.25 M in CH_3CN) was used as a coupling agent for the unmodified RNA phosphoramidites and 5-ethylthiotetrazole (0.25 M in CH_3CN) was used for **1**. The coupling time was 7 min for the unmodified RNA phosphoramidites while **1** was coupled for 10 min. The RNAs were deprotected and cleaved from the resin in a 1:1 solution (2 mL) of CH_3NH_2 (8 M in EtOH) and satd. aq. NH_3 at 65 °C for 1 h. The solvent was removed *in vacuo* and the 2′O-TBDMS groups were removed by incubation in a solution of $Et_3N{\cdot}3HF$ (300 μL) in DMF (100 μL) at 55 °C for 1.5 h, followed by addition of deionized and sterilized water (100 μL). This solution was transferred to a 50 mL Falcon tube, *n*-butanol (20 mL) was added, the mixture stored at −20 °C for 12 h, centrifuged (4000 rpm) at 4 °C for 1 h, the solvent decanted from the RNA pellet and the pellet dried *in vacuo*.

1.2 Oligonucleotide purification

The RNA oligonucleotides were subsequently purified by 20% DPAGE and extracted from the gel slices using the “crush and soak method” with Tris buffer (250 mM NaCl, 10 mM Tris, 1 mM Na_2EDTA, pH 7.5). The solution was filtered through GD/X syringe filters (0.45 μm, 25 mm diameter, Whatman, USA) and was subsequently desalted using Sep-Pak cartridges (Waters, USA), following the instructions provided by the manufacturer. The dried oligonucleotides were dissolved in deionized and sterilized water (200 μL). The concentration of the oligonucleotides was determined by measuring absorbance at 260 nm using a Perkin Elmer Inc. Lambda 25 UV/Vis spectrometer and calculated by Beer′s law. Mass spectrometric analyses of the **Çm**-labeled oligonucleotides were performed on an HRMS (ESI) (Bruker, MicrOTOF-Q) in negative ion mode.

1.3 Analysis of spin-labeled oligonucleotides by MS and EPR spectroscopy

The incorporation of **Çm** into the TMR aptamer (**Table S1**) was confirmed by HRMS (ESI) analysis; its calculated and observed monoisotopic mass is listed in **Table S1**. The amount of radical in the oligonucleotide was determined by spin counting using EPR spectroscopy (**Table S1**).

Table S1. Sequences of the **Çm**-labeled TMR aptamers and their monoisotopic masses.

Name	Sequence	calc. mass	meas. mass	radical %
C12C30	5'-GGA-CGA-CUG-AA**Çm**-CGA-AAG-GUU-CUU-GGC-UG**Çm**-UUC-GGC-AGA-GGU-ACG-UCC-3'	15893.28	15893.60	90
C12C47	5'-GGA-CGA-CUG-AA**Çm**-CGA-AAG-GUU-CUU-GGC-UGC-UUC-GGC-AGA-GGU-ACG-U**Çm**C-3'	15893.28	15893.60	88
C30C47	5'-GGA CGA CUG AAC CGA AAG GUU CUU GGC UG**Çm** UUC GGC AGA GGU ACG U**Çm**C-3'	15893.28	15918.39	95

1.4 EPR sample preparation

The purified oligonucleotides were dissolved in glutamate buffer (130 mM, pH 7.5, 10 mM NaCl). For the samples which were recorded in the presence of the ligand, 175 µM 5-TAMRA were added to the buffer. 20% d_6-ethylene glycol was added to the aqueous buffer solutions before transferring the samples into the respective sample tubes. The concentration of the RNA was 100 µM. In the sample including the ligand, the final concentration of 5-TAMRA was 140 µM. For X-band measurements, 20 µL were transferred into 2.8 mm (outer diameter) Suprasil tubes. For Q-band measurements, 10 µL were transferred into 1.6 mm (outer diameter) Suprasil tubes. For G-band measurements, the sample was transferred to 0.55 mm (outer diameter; 0.45 mm inner diameter) quartz capillaries. The active volume of the G-band samples is approximately 300 nL.

2 Experimental details of EPR experiments

2.1 X-band PELDOR

The X-band (9.5 GHz, 0.3 T) PELDOR experiments were conducted on a custom-built X-band spectrometer.[2] This spectrometer employs a BRUKER ER41118X-MS3 split-ring resonator, a continuous-flow helium cryostat (CF935, Oxford Instruments) and a temperature control system (ITC 502, Oxford Instruments). Microwave pulses were generated via a MW synthesizer (HP 8672A), modulated by two arbitrary waveform generators (AWG, SD devices SDR14), and amplified using a 1 kW traveling-wave tube amplifier (TWT, Applied Systems Engineering 117X). All measurements at X-band were performed at 50 K.

The 4-pulse PELDOR sequence was used for the experiments,[3],[4] incorporating a 4-step phase cycle (0°, 90°, 180°, 270°) for the pump pulse, with an additional phase cycle (x, -x) for the detection pulses.[5] Gaussian-shaped pulses were used in all measurements,[6] with the full width at half maximum (FWHM) for detection pulses set to 23.4 ns (72 ns total length) and for the pump pulse to 8.78 ns (28 ns total length). The amplitudes were optimized for each measurement, and the pulse bandwidths were comparable to rectangular pulses with respective lengths of 32 ns and 12 ns. The interpulse delay τ_1 was set to 448 ns and τ_2 was set to 3500 ns.

To minimize the electron spin echo envelope modulation (ESEEM) effect from ^{2}H nuclei in the solvent, τ_1 averaging was implemented, with τ_1 incremented in 56 ns steps across 8 increments.[7] The echo was integrated over 56 ns at peak intensity. The pump pulse was set to the maximum of the nitroxide spectrum, while the detection sequence used a frequency offset. For each sample, six time traces were recorded with frequency offsets ranging from 40 to 90 MHz in 10 MHz steps, as shown in Figure S1 (left).

2.2 Q-band PELDOR

The Q-band (33 GHz, 1.2 T) PELDOR measurements were recorded on a Bruker ELEXSYS E580 pulsed X/Q-band EPR spectrometer equipped with a Bruker EN 5107-D2 resonator, a PELDOR unit (Bruker E580-400U), an AWG (SpinJet-AWG), a continuous-flow helium cryostat (Oxford Instruments CF935), and a temperature control system (Oxford Instruments ITC 502). The microwave pulses were amplified by a 150 W Bruker AmpQ TWT (Applied Systems Engineering Inc. 187Ka). All measurements were carried out at 50 K.

For the PELDOR experiments, the 4-pulse PELDOR sequence was used.[3],[4] A 4-step phase cycle (0°, 90°, 180°, 270°) of the pump pulse and a 2-step phase cycle of the detection pulses (x, -x) were performed.[5] The measurements of C12C30 and C12C47 were performed with Gaussian shaped pulses.[6] For the observer sequence, a pulse length of 52 ns was used for the π/2 pulses and 104 ns for the π pulses. A 28 ns pump pulse was used. For the measurements of C30C47, rectangular pulses were used. For the detection sequence a pulse length of 32 ns was used and a length of 12 ns for the pump pulse. The amplitude was set accordingly. The echo was integrated for 60 ns around the maximum intensity. The initial τ_1 value was chosen to be 232 ns and was incremented in 8 steps of 16 ns to account for the ^{2}H ESEEM.[7] The time τ_2 was set to 5 µs. The position of the pump pulse was set to the maximum of the nitroxide spectrum, and the detection sequence was applied at a frequency offset of -80 MHz (see Figure S1, center).

2.3 G-band PELDOR

All G-band (180 GHz, 6.4 T) PELDOR experiments were carried out on a custom-built G-band spectrometer.[8] All measurements were carried out at 50 K.

For all experiments, the 4-pulse PELDOR sequence[3],[4] was used with rectangular pulses. The pulse lengths were optimized with a constant set amplitude. The π/2 pulses were around 46 ns long and the detection π pulses were around 72 ns long. The π pump pulse was always set slightly shorter than the detection π pulse (around 70 ns). The interpulse delay τ_1 was set to 220 ns. The time τ_2 was adjusted in such a way that the entire dipolar oscillation was recorded. The detection and pump sequences were applied at a constant offset of 60 MHz with the pump pulse always set at higher frequencies. The field positions were varied to record a set of five time traces. Traces were recorded at the resonance field positions of the principal g-values (positions called xx, yy, zz). Additionally, two traces were recorded in the middle between xx and yy (position xy) and between yy and zz (position yz) (see Figure S1, right).

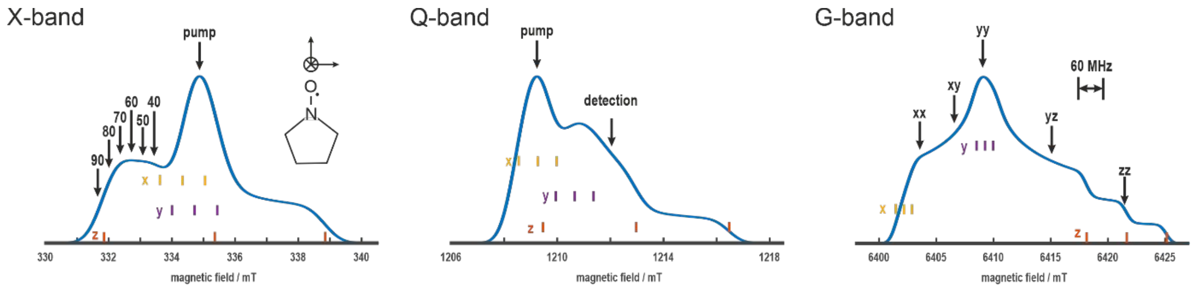

Figure S1: Simulated powder spectra using Easyspin[9] of a typical nitroxide radical (g_{xx}=2.0088, g_{yy}=2.0061, g_{zz}=2.0027 and A_{xx}=A_{yy}=20 MHz, A_{zz}=90 MHz)[10] at X- (9.5 GHz, 0.3 T; left), Q- (34 GHz, 1.2 T; center) and G-band (180 MHz, 6.4 T; right). The resonance field positions for orientations of the external magnetic field parallel to the principal axes of the magnetic tensors are depicted by the colored lines (the central ones corresponding to the g value resonances). The positions of the pulses used for orientation-selective PELDOR experiments are shown. The frequency offsets at X-band are given in MHz. The insert shows the definition of the coordinate system of a nitroxide spin label.

3 Additional experimental data

3.1 Primary and background-corrected Q-band PELDOR data

Figure S2 shows the recorded PELDOR time traces which were acquired at Q-band. The data of all three spin labeled constructs of the TMR-3 aptamer are shown in absence (black) and presence (magenta) of the ligand 5-TAMRA. The background functions are shown for each of the primary data. A dimensionality d=3 was used for all datasets except for C12C30 (with and without 5-TAMRA; d=2.5). During data processing and analysis, we noticed that d=3 yields an unrealistically long distance for C12C30 which could not be rationalized. Smaller distances were not affected by a change of the background dimensionality

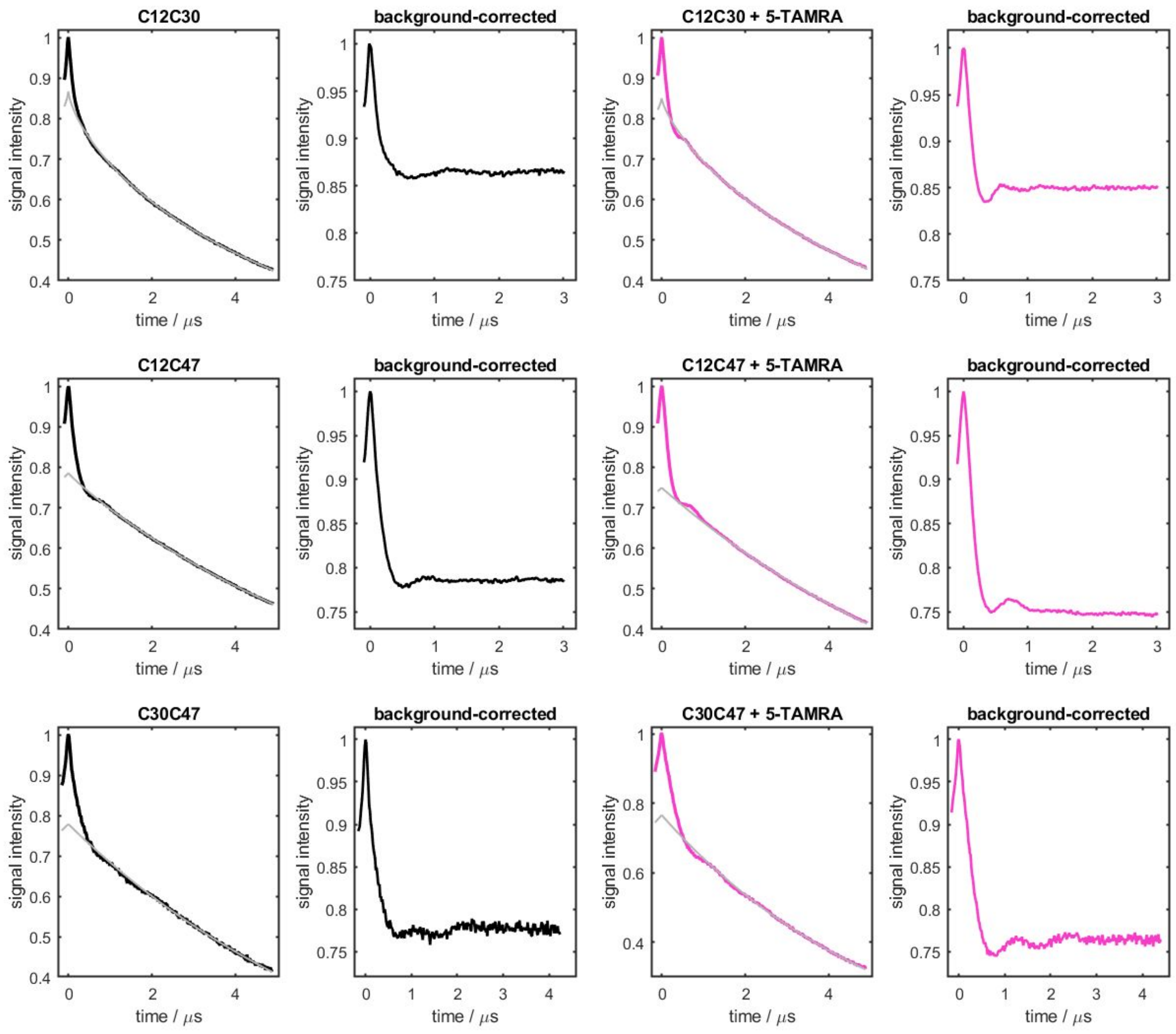

Figure S2: Experimental Q-band PELDOR data of the spin labeled TMR-3 aptamer in presence (magenta) and absence (black) of 5-TAMRA. Each of the primary data are shown with the respective background function. The background-corrected data are also shown. The background correction was performed using DeerAnalysis 2022.[11]

3.2 C30C47+5-TAMRA – different sample compositions

Q-band PELDOR data for C30C47 in presence of 5-TAMRA were recorded for different sample compositions to try to minimize the amount of unbound RNA in the sample. Figure S3 shows the resulting distance distributions from the different samples. The first sample that was prepared with only 1.5-fold excess of 5-TAMRA to RNA is shown in black. Two samples were prepared with a larger excess of 5-TAMRA (around 3-fold, pink and red). In these datasets, the distance contribution around 2.4 nm is slightly smaller than in the first sample. This is also true, when considering the uncertainty of the background correction (not shown).

Since the TMR-3 aptamer was originally selected in the presence of a low concentration of Mg^{2+} ions, a last sample with 1 mM Mg^{2+} and 3-fold excess 5-TAMRA was prepared (yellow). This also did not reduce the additional distance contributions. We concluded that the solubility of 5-TAMRA, particularly around the freezing point, and the potential of a higher KD value after introducing the **Çm** spin labels could cause the incomplete binding of TMR-3 to 5-TAMRA in our samples.

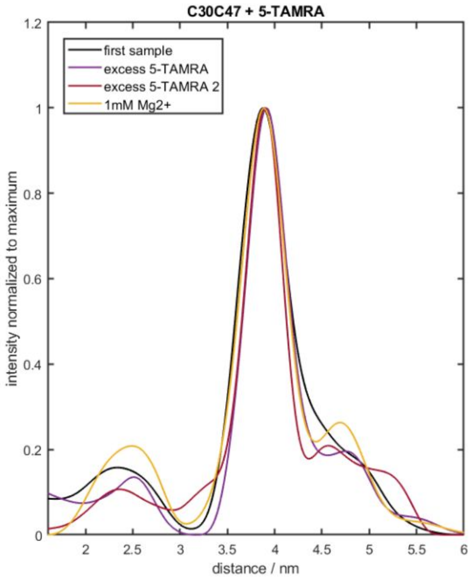

Figure S3: Distance distributions obtained for C30C47 in presence of 5-TAMRA at Q-band. The results from different sample compositions are shown. The first sample (black) contained a 1.5-fold excess of 5-TAMRA with respect to the RNA. Two samples with around 3-fold excess of 5-TAMRA were prepared as well (purple and red). One sample was prepared with 1 mM Mg^{2+} ions (yellow).

3.3 Background-corrected experimental orientation-selective PELDOR data

The full set of background-corrected orientation-selective PELDOR time traces recorded at X-band (Figure S4) and G-band (Figure S5) is shown. The offsets between the detection and the pump sequences are indicated for the X-band data and the field positions where the data was recorded is indicated for the G-band data (see Figure S1 for an explanation of the positions).

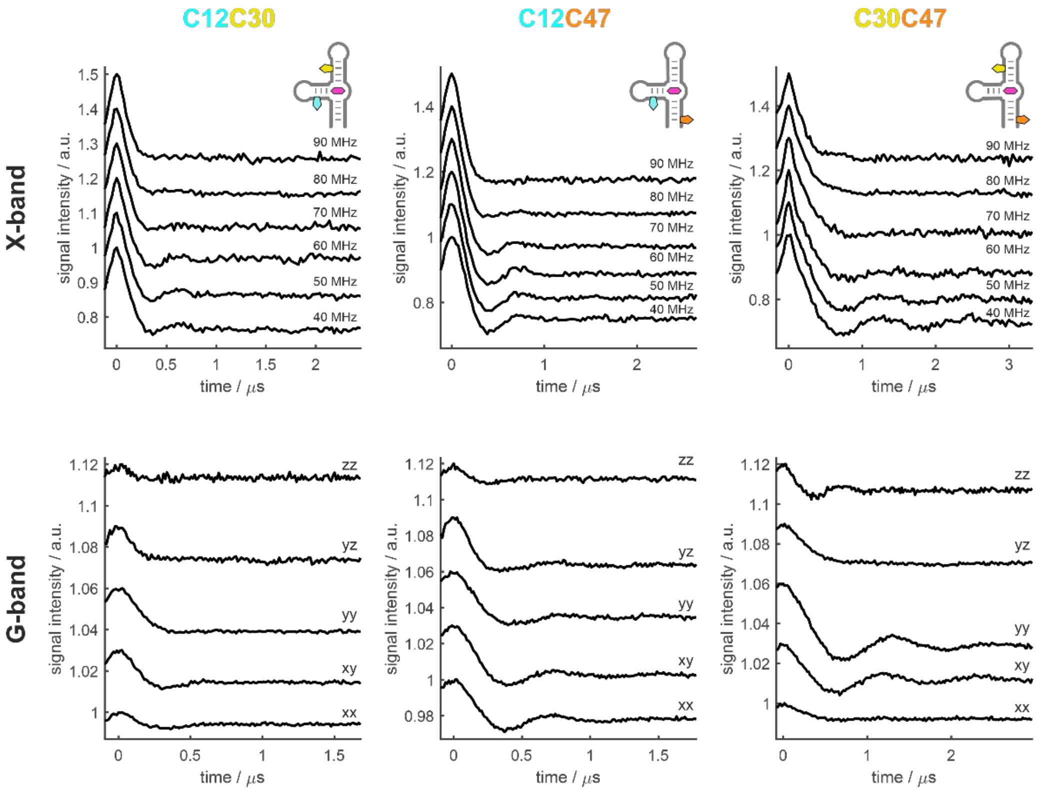

Figure S4: Background-corrected orientation-selective PELDOR data of the three ***Çm*** *spin-labeled constructs of the TMR-3 aptamer in the presence of the ligand 5-TAMRA, measured at X-band (top row; 9.4 GHz, 0.3 T) and G-band (bottom row; 180 GHz, 6.4 T). All time traces are shown with an offset to improve readability.*

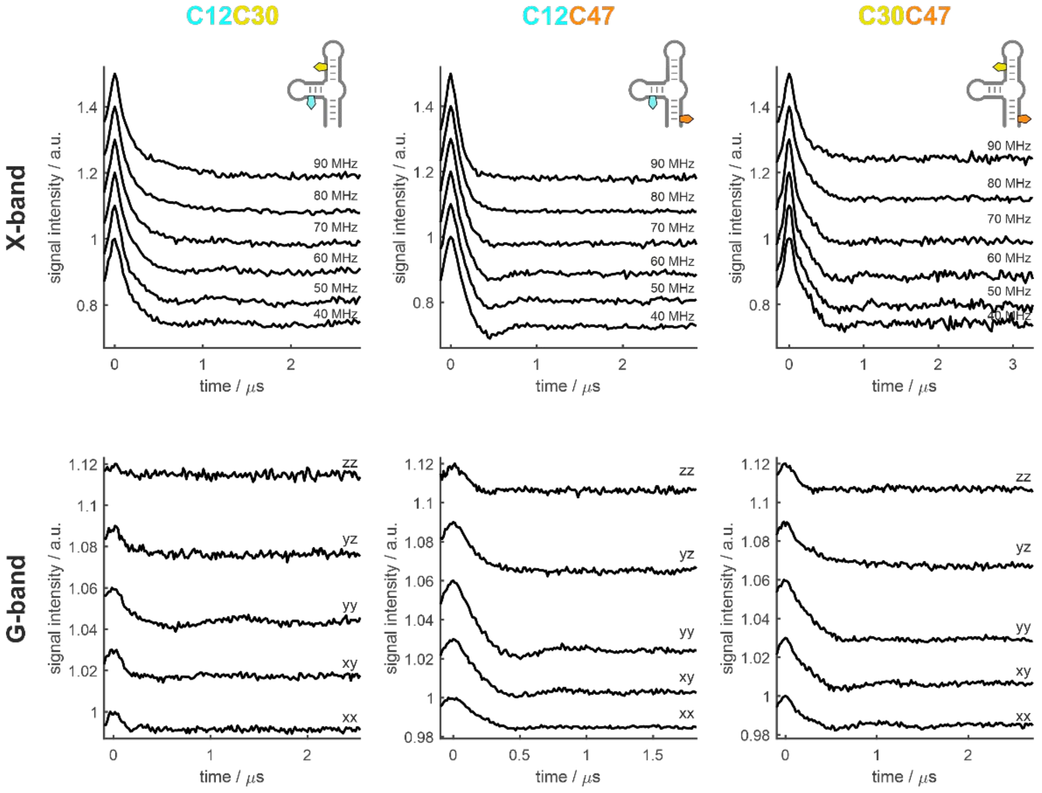

Figure S5: Background-corrected orientation-selective PELDOR data of the three ***Çm*** *spin-labeled constructs of the TMR-3 aptamer in the absence of the ligand 5-TAMRA, measured at X-band (top row; 9.4 GHz, 0.3 T) and G-band (bottom row; 180 GHz, 6.4 T). All time traces are shown with an offset to improve readability.*

Figure S6 shows a simplified schematic of the orientation of two parallel spin labels. This represents, e.g., a helix geometry such as the continuous helix stack P1/P3 in the TMR-3 aptamer. The principal axis systems of the nitroxides are shown. The x-axis is along the N-O bond and the z-axis is the normal vector of the plane of the spin label's ring system. The z-axes are collinear. Because the aminoxyl moiety is close to the helical axis, the z-axes are also almost collinear to the helical axis and the interspin vector R.

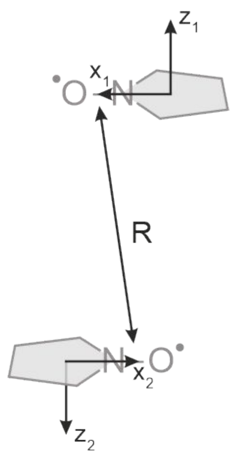

Figure S6: Simplified schematic that represents the orientation of two parallel nitroxide radicals. The respective coordinate axes are shown (x1/2 and z1/2). The y-axes can be calculated by forming the cross product of x and z. The interspin vector R is shown as well.

4 Additional simulated & fitting data

Figure S7 shows a comparison of the experimental and simulated X- and G-band data of the three spin labeled constructs of TMR-3 in complex with its ligand 5-TAMRA. The simulations were performed using the 20 published NMR structures (pdb: 6GZR & 6GZK). It can clearly be seen that the dampening of the oscillations in the simulated data is not sufficient to represent the experiments. This can be attributed to the very narrow distribution of distances in 20 low RMSD NMR structures (Figure S8).

Figure S8 shows the distribution width of the bundle of 20 NMR structures (green, pdb: 6GZR & 6GZK). It also contains the distribution of distances from around 1000 structures of the ensemble of structures which were generated using the published NMR restraints and the measured EPR distance widths (orange). Only the distances of 1000 structures with a target function value that increases by 10% with respect to the lowest value in the ensemble are shown.

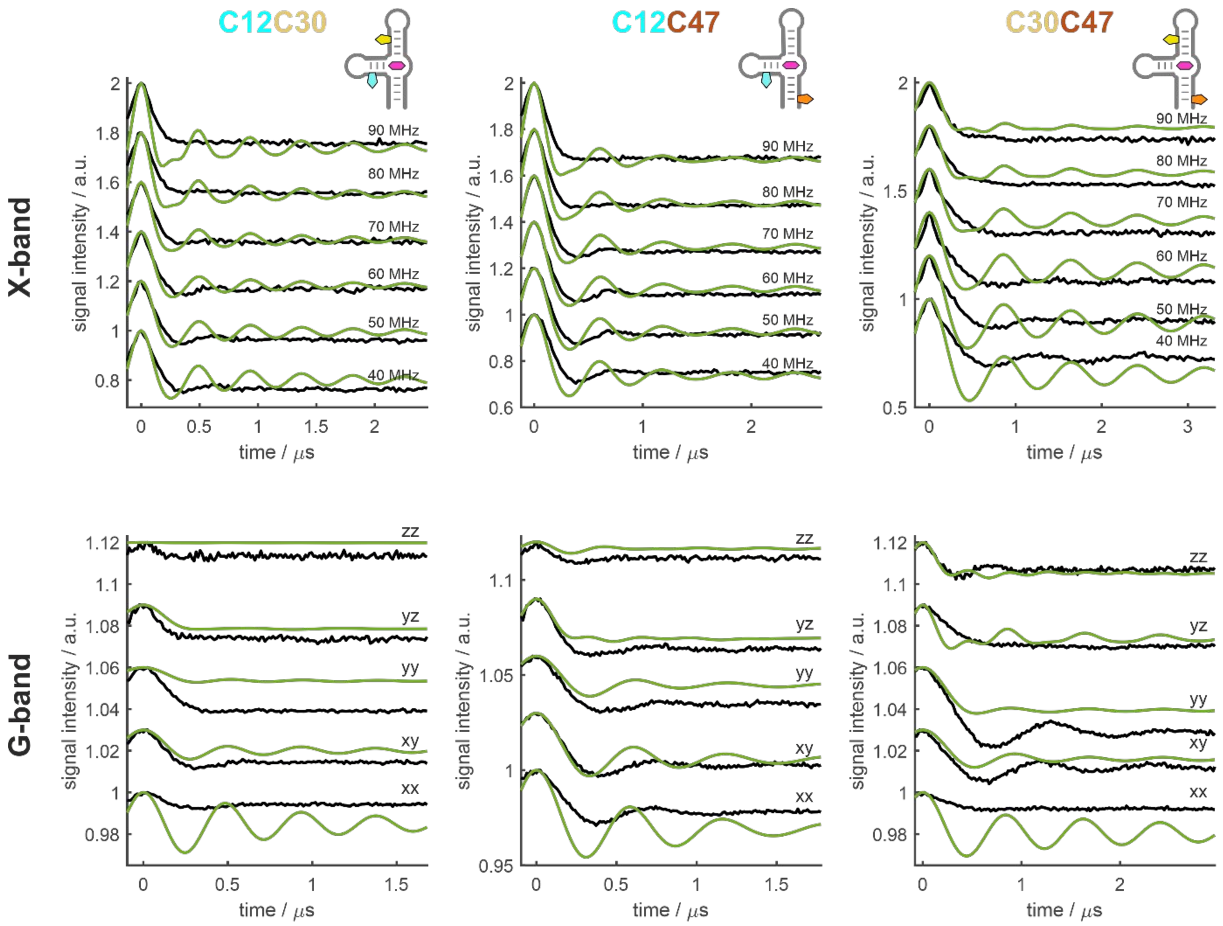

Figure S7: Experimental orientation-selective PELDOR data (black) of the three ***Çm*** *spin-labeled constructs of the TMR-3 aptamer in the presence of the ligand, measured at X-band (top row; 9.4 GHz, 0.3 T) and G-band (bottom row; 180 GHz, 6.4 T). In addition, a simulation is shown (green). The spin label orientations used in the simulations were obtained by overlaying the structure of* ***Çm*** *onto the respective nucleotide in the NMR structures of the TMR-3/5-TAMRA complex (pdb 6GZR & 6GZK).[12] All time traces are shown with an offset to improve readability.*

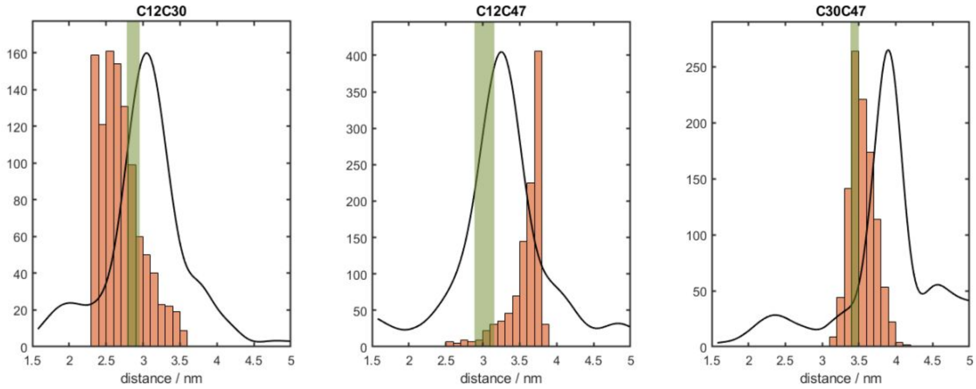

Figure S8: Experimental distance distributions of the three ***Çm*** *spin-labeled constructs of the TMR-3 aptamer in the presence of the ligand, obtained at Q-band, are shown (black). The green shaded areas indicate the distances obtained by applying the structure of* ***Çm*** *to the published NMR structures of the TMR-3/5-TAMRA complex (pdb: 6GZR & 6GZK).[12] Additionally, the distance distribution of 1000 structures with the lowest target function values from the structure ensemble of the TMR-3/5-TAMRA complex is shown (orange). The ensemble was generated by combining NMR and EPR restraints. 50 of these 1000 structures were used for obtaining the fit in Figure 4 of the main text (orange).*

Figure S9 shows a comparison of the experimental X- and G-band data of the three spin labeled constructs of TMR-3 in complex with 5-TAMRA and the results from the fitting

procedure (purple). In this case, a cut-off of the target function was chosen for the large ensemble such that around 8500 structures remained (cut-off 40 Å^2, see Figure S10). For the fit, 50 structures were selected that best represent the experimental data. The quality of the fit is very good. It is slightly surpassed by the fit that is presented in the main text, where 10 conformers from the ensemble of the fit of the free RNA were added to the data. This improved the fit quality of C30C47, particularly at small times t. Figure S11 shows the comparison of the root mean square deviation (RMSD) of the two fits that are presented in the main text and the fit that is presented in Figure S9. The orange bar shows the RMSD of the fit where only 1000 structures with the lowest target function values were available. The purple bar shows the RMSD of the fit presented in Figure S9 where a target function cut-off of 40 Å^2 was chosen (8500 structures available). The blue bar shows the RMSD of the fit where in addition to choosing from the 8500 structures of the bound state, 10 structures from the fit of the free RNA were added after the fourth iteration step of the fit. The improvement from the fit from 1000 structures to the other two fits is almost two-fold which can in large part be attributed to a better fit of the data of C30C47. The contributions of the individual datasets to the total RMSD are shown in Figure S11 (right). The improvement of the RMSD that is achieved by adding 10 conformers from the fit of the unbound state is not dramatic. However, the visual improvement of the fit of C30C47 is noticeable and the distribution of distance in the conformers that were selected by the fit becomes significantly smoother when the unbound state is added after the fourth fit iteration (not shown).

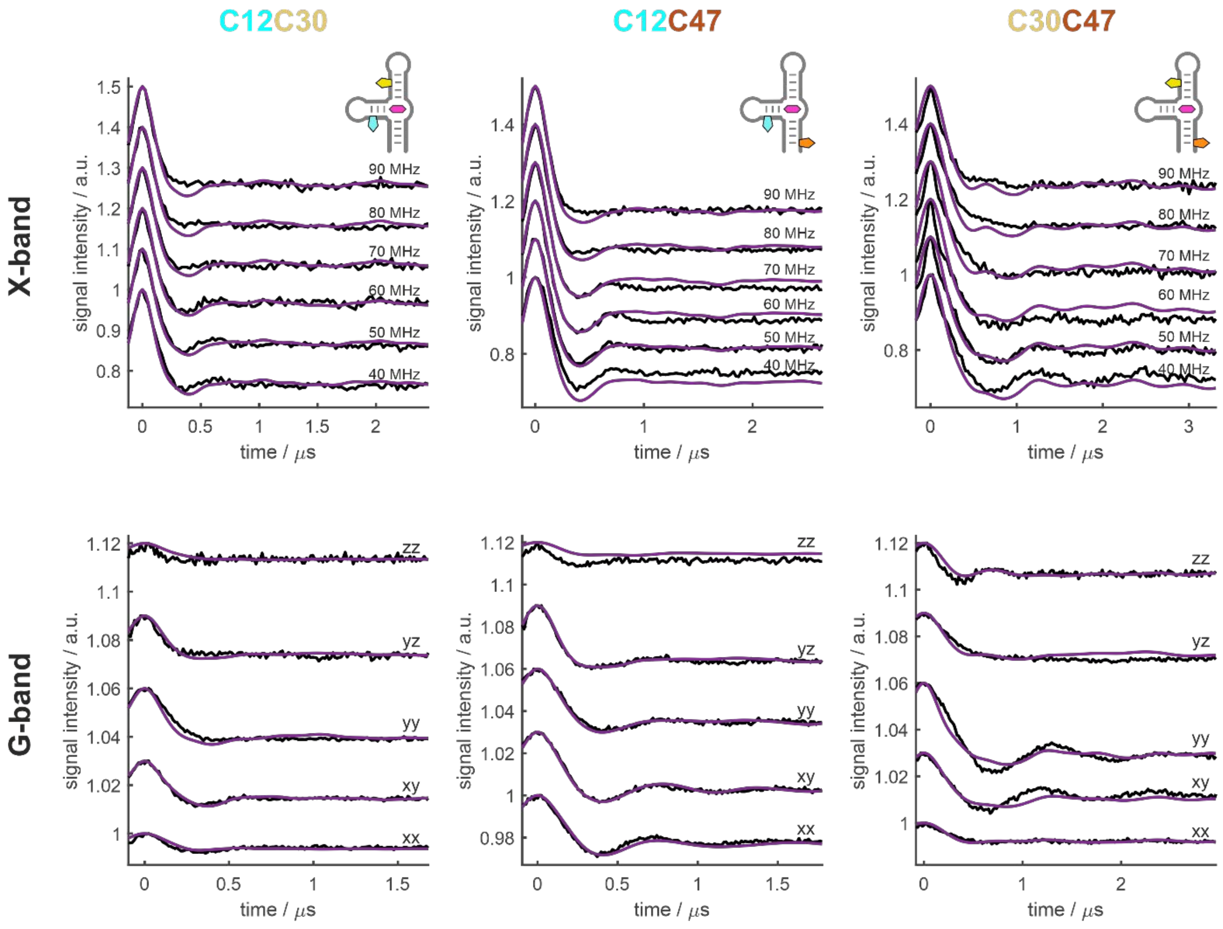

*Figure S9: The experimental orientation-selective PELDOR data (black) of the three **Çm** spin-labeled constructs of the TMR-3 aptamer in the presence of the ligand, measured at X-band (top row; 9.4 GHz, 0.3 T) and G-band (bottom row; 180 GHz, 6.4 T) are shown. They are compared to the result of a fit which selected 50 structures from an ensemble of 8500 structures, generated by combining NMR and EPR restraints of the TMR-3/5-TAMRA complex (purple). The time traces are shown with an offset to improve readability.*

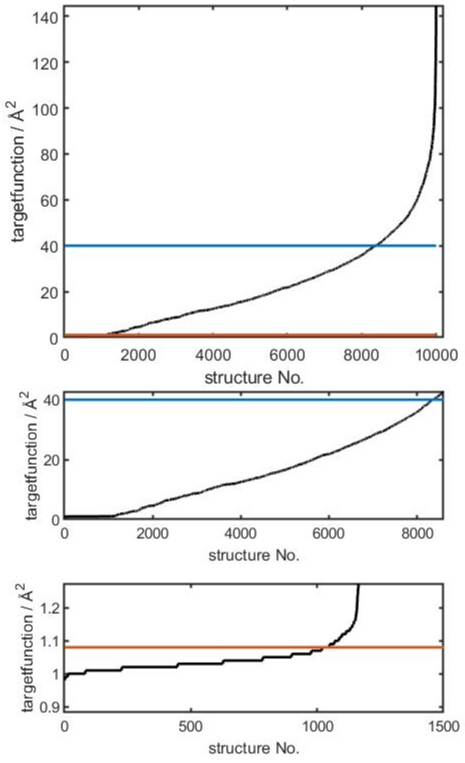

Figure S10: Target function (TF) values obtained for the bundle of the RNA-ligand complex (black). The cut-off at 40 Å^2 is shown in blue. Around 8,500 structures fall into this part of the bundle and were used in the blue fit in Figure 3 of the main text and purple fit in Figure S9. This section of the TF curve is magnified in the center plot. An additional cut-off is shown at 1.08 Å^2. This part of the bundle includes around 1,000 structures and were used for the orange fit in Figure 3 of the main text. This section of the TF curve is magnified in the bottom plot.

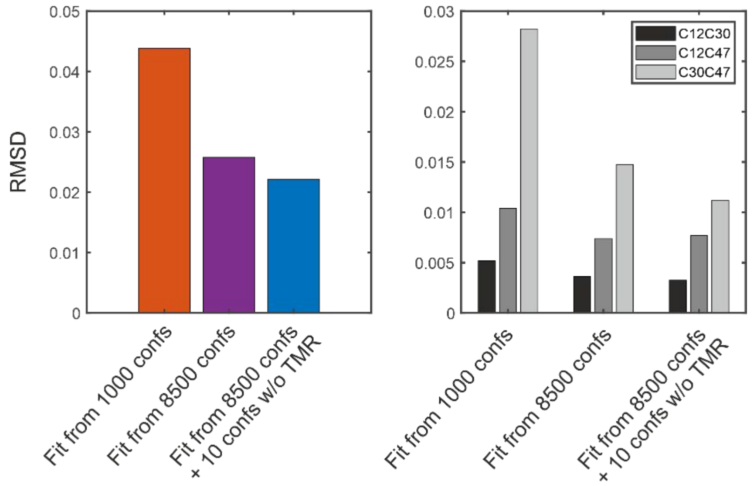

Figure S11: RMSD values obtained for different fits of the orientation-selective PELDOR data of the TMR-3/5-TAMRA complex. The left plot shows the total value of the RMSD and the right plot shows the individual contributions of the three spin labeled constructs for each fit. Orange: Result from the fit which selected 50 conformers from a sub-ensemble of 1000 structures (orange fit in Figure 3 of the main text). Purple: Result from the fit which selected 50 conformers from a sub-ensemble of 8500 structures (target function cut-off: 40 Å^2; purple fit in Figure S9). Blue: Result from the fit which selected 50 conformers from a sub-ensemble of 8500 structures. Here, 10 structures with R(C30C47)<3 nm from the fit of the unbound state were added after the fourth iteration of the fit (blue fit in Figure 3 of the main text).

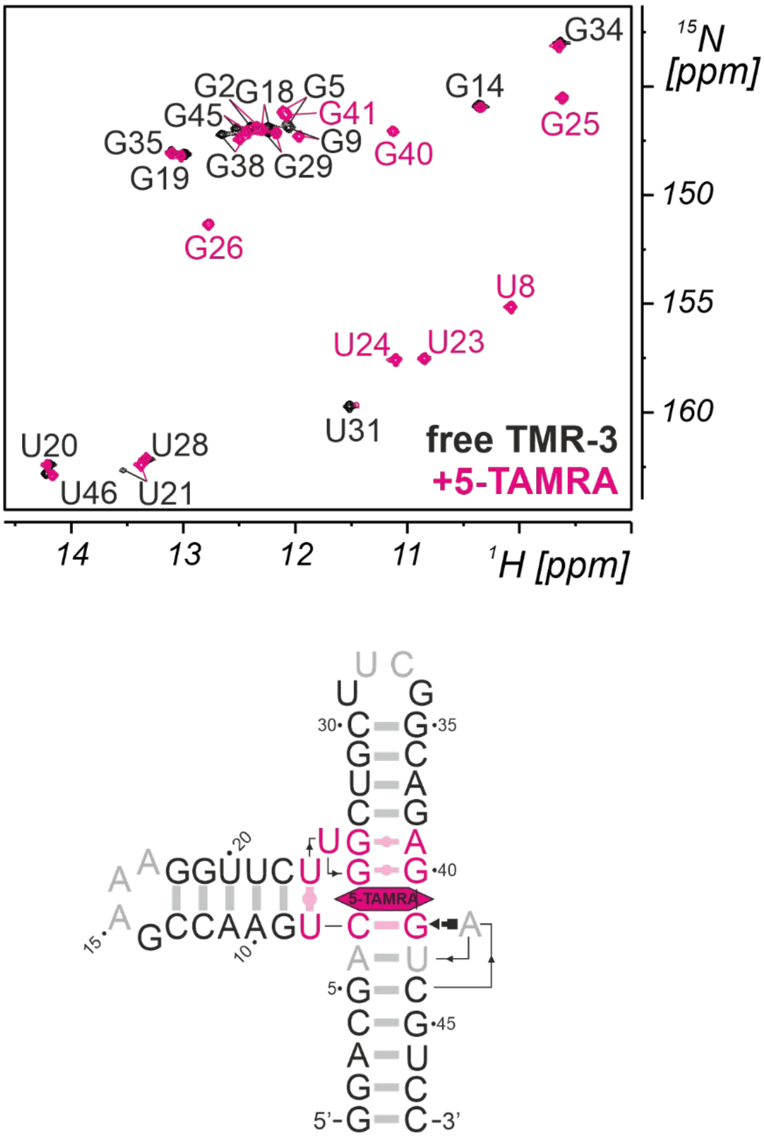

Figure S12: Base pairing pattern of the free TMR-3 aptamer compared to TMR-3 in the 5-TAMRA complex. Top: Overlay between the imino-group region of 1H,15N-HSQC spectra of the free aptamer (black) and the aptamer in the 5-TAMRA complex (magenta). Resonance assignments are given in black for imino group signals, which are present both in the free and the bound state. Resonances observed only for the ligand-complex are annotated in magenta. Bottom: Schematic representation of the TMR-3/5-TAMRA complex. Residues for which imino group resonances are observed only in the complex and their base pairing partners are highlighted in magenta, those, for which imino resonances are observed in both the free and the bound state and their base pairing partners are shown in black. All other residues are grey. The ligand 5-TAMRA is indicated.

Figure S13 shows 20 structures of the TMR-3/5-TAMRA complex. These were selected in the first 20 iterations of the fitting procedure that included 10 unbound state structures. Only the structures of the ligand-bound state are shown. The structures were aligned by minimizing the heavy atom RMSD of helices P1 (orange) and P3 (yellow). It can be seen that even the aligned helices which form a continuous stack with the ligand 5-TAMRA (magenta) sandwiched between them, show significant conformational variety. The helix P2 is even more flexible, covering a large range of conformations.

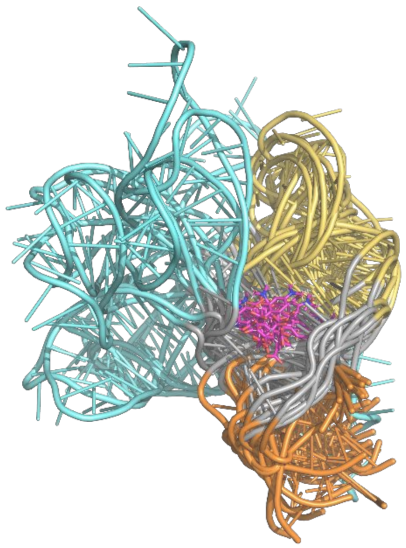

Figure S13: 20 structures of the TMR-3/5-TAMRA complex are shown in cartoon representation. These are obtained from the first 20 iterations of the fit of the orientation-selective PELDOR data of the TMR-3/5-TAMRA complex which selected 50 structures from a sub-ensemble of 8500 structures with additional 10 structures from the fit of the unbound state which were added after the fourth iteration. The helix P1 is shown in orange, P2 in cyan and P3 in yellow. 5-TAMRA is shown in magenta. The ***Çm*** *spin label structures are hidden by the cartoon representation. All structures were aligned by minimizing the heavy atom RMSD of helices P1 and P3.*

Figure S14 shows the distributions of the distances and the angles between the z-axes (zz angle) of both spin labels or the x-axes (xx angle) of both spin labels. These values were obtained from the conformers that were selected by the fit of the X- and G-band data of the TMR-3/5-TAMRA complex. They are extracted from the fit that included 10 conformers of the unbound state and 50 conformers from 8500 possible conformers of the aptamer-ligand

complex. Additionally, the values that are obtained when overlaying the structure of **Çm** onto the respective nucleotides in the 20 published NMR structures are shown (green).

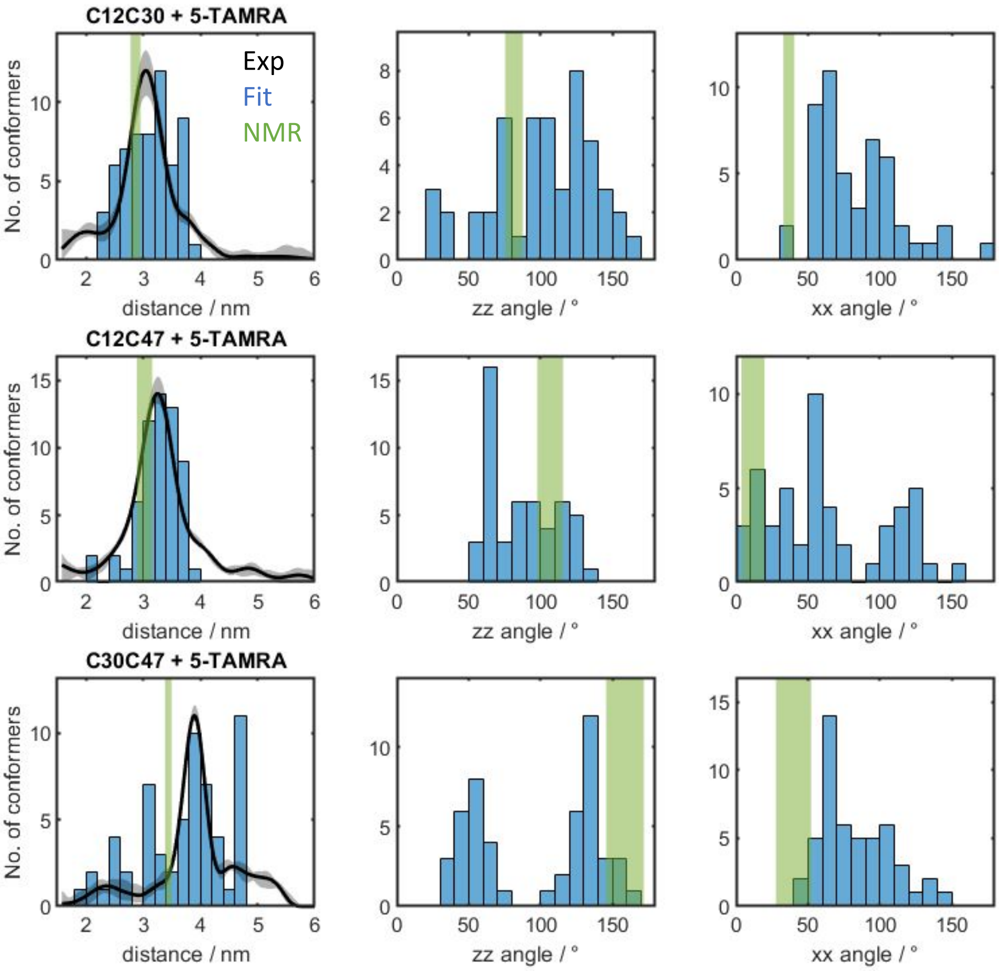

Figure S14: The distributions of the interspin distance, the angle between the z-axes of the spin labels (zz angle) and the angle between the x-axes of the spin labels (xx angle) are shown (blue). These were obtained from the fit of the experimental data of the TMR-3/5-TAMRA complex. The fit selected 50 structures from an ensemble of 8500 structures with additional 10 structures from the fit of the free TMR-3 data which were added after the fourth iteration of the fit. As a guidance, the experimental distance distribution is shown (black, left). The distribution which are obtained when applying the structure of the ***Çm*** *spin label onto the published NMR structures (pdb 6GZR & 6GZK)*[12] *are shown as a comparison.*

References

[1] H. Y. Juliusson; A. L. J. Segler; S. T. Sigurdsson. *European J. Org. Chem.* **2019**, *2019* (23), 3799–3805.

[2] M. Bretschneider; P. E. Spindler; O. Y. Rogozhnikova; D. V. Trukhin; B. Endeward; A. A. Kuzhelev; E. Bagryanskaya; V. M. Tormyshev; T. F. Prisner. *J. Phys. Chem. Lett.* **2020**, *11* (15), 6286–6290.

[3] R. E. Martin; M. Pannier; F. Diederich; V. Gramlich; M. Hubrich; H. W. Spiess. *Angew. Chemie Int. Ed.* **1998**, *37* (20), 2833–2837.

[4] M. Pannier; S. Veit; A. Godt; G. Jeschke; H. . Spiess. *J. Magn. Reson.* **2000**, *142* (2),

331–340.

[5] C. E. Tait; S. Stoll. *Phys. Chem. Chem. Phys.* **2016**, *18* (27), 18470–18485.

[6] M. Teucher; E. Bordignon. *J. Magn. Reson.* **2018**, *296*, 103–111.

[7] G. Jeschke. *Annu. Rev. Phys. Chem.* **2012**, *63*, 419–446.

[8] M. M. Hertel; V. P. Denysenkov; M. Bennati; T. F. Prisner. *Magn. Reson. Chem.* **2005**, *43* (S1), S248–S255.

[9] S. Stoll; A. Schweiger. *J. Magn. Reson.* **2006**, *178* (1), 42–55.

[10] D. J. Schneider. Berliner, L., Reuben, J., Eds.; Plenum Press, 1989.

[11] G. Jeschke; V. Chechik; P. Ionita; A. Godt; H. Zimmermann; J. Banham; C. R. Timmel; D. Hilger; H. Jung. *Appl. Magn. Reson.* **2006**, *30* (3–4), 473–498.

[12] E. Duchardt-Ferner; M. Juen; B. Bourgeois; T. Madl; C. Kreutz; O. Ohlenschläger; J. Wöhnert. *Nucleic Acids Res.* **2020**, *48* (2), 949–961.
